# *S*-adenosyl-L-homocysteine triggers an adaptive response that extends lifespan through the *YBT1* ABC transporter

**DOI:** 10.1073/pnas.2621286123

**Published:** 2026-10-16

**Authors:** Koji Masumura, Sayaka Kawasaki, Mayu Tsutsumi, Hiiro Yamashita, Muneyoshi Kanai, Haruto Nakajo, Wakaba Yagi, Shoma Katsunoi, Kaori Yoneyama, Miyuki Kawano-Kawada, Takayuki Sekito, Takafumi Ogawa, Tomoyoshi Soga, Masaki Mizunuma

**Affiliations:** ^a^ Laboratory of Healthy Aging, Unit of Biotechnology, Graduate School of Integrated Sciences for Life, Hiroshima University, Higashi-Hiroshima 739-8530, Japan; ^b^ Brewing Microbiology Division, National Research Institute of Brewing, Higashi-Hiroshima 739-0046, Japan; ^c^Laboratory of Molecular Physiology and Genetics, Faculty of Agriculture, Graduate School of Agriculture, Ehime University, Matsuyama 790-8566, Japan; ^d^ Research and Development Bureau, Saitama University, Saitama 338-8570, Japan; ^e^ Division of Cell-Free Sciences, Proteo-Science Center, Ehime University, Matsuyama 790-8577, Japan; ^f^ Division of Genetic Research Support, the Advanced Research Support Center, Ehime University, Matsuyama 790-8566, Japan; ^g^ Institute for Advanced Biosciences, Keio University, Tsuruoka 997-0052, Japan; ^h^ Human Biology-Microbiome-Quantum Research Center, Keio University, Tokyo 160-8582, Japan; ^i^ Hiroshima Research Center for Healthy Aging, Hiroshima University, Higashi-Hiroshima 739-8530, Japan

**Keywords:** *S*-adenosyl-L-homocysteine, mitochondria, TORC1, ABC transporter, chronological lifespan

## Abstract

*S*-adenosyl-L-methionine (SAM) is a fundamental methyl donor; however, the physiological role of its byproduct and competitive inhibitor, *S*-adenosyl-L-homocysteine (SAH), remains unclear. Here, we show that transient reactive oxygen species (ROS) production triggered by SAH during the early logarithmic phase mimics partial SAH hydrolase inhibition, extending the yeast chronological lifespan (CLS) through a hormetic mechanism. This study reveals that the dual nature of ROS in aging is determined by the specific degree of SAH hydrolase activity. We identified the adenosine 5’-triphosphate-binding cassette (ABC) transporter Ybt1 as a key downstream effector of SAH. Ybt1 localizes to the vacuolar membrane and facilitates the sequestration of SAM from the cytosol into the vacuole. Notably, *YBT1* overexpression extends CLS, mimicking SAH effects through an integrated pathway involving target of rapamycin complex 1 downregulation, autophagy, and adenosine 5’-monophosphate-activated protein kinase activation. Our findings establish that SAH acts as a metabolic signal coupling nutrient and energy availability to longevity via Ybt1, defining an adaptive/hormetic response for lifespan regulation in yeast.

## Significance

Although the dualistic nature of reactive oxygen species (ROS) in cellular health is well recognized, the precise mechanisms controlling this delicate balance remain elusive. We address this fundamental gap by demonstrating that S-adenosyl-L-homocysteine (SAH) acts as an endogenous metabolic signal that dictates a hormetic threshold for ROS, converting ROS from deleterious agents to vital longevity signals. Mechanistically, we uncovered a role for the adenosine 5’-triphosphate (ATP)-binding cassette (ABC) transporter Ybt1; it mediates vacuolar S-adenosyl-L-methionine (SAM) sequestration to modulate the target of rapamycin complex 1 (TORC1) and activate adenosine 5’-monophosphate (AMP)-activated protein kinase (AMPK), thereby optimizing mitochondrial respiration in yeast. This study establishes a conserved evolutionary paradigm that couples nutrient availability, redox homeostasis, and organismal aging.

Mitochondrial function and reactive oxygen species (ROS) production are linked to the hormetic response and aging processes (1–4). ROS play a dual role, acting as both harmful agents and crucial signaling molecules (3–5). This is central to hormesis, where mild stress-induced increases in ROS trigger beneficial adaptive responses, specifically termed mitohormesis when rooted in mitochondrial signaling (2–8). Studies in *Saccharomyces cerevisiae* have shown that enhanced mitochondrial respiration and elevated ROS levels under glucose restriction or target of rapamycin complex 1 (TORC1) deficiency (*tor1*Δ) can extend lifespan (9–12). This challenges traditional views on oxidative stress and aging, highlighting the complexity of cellular redox homeostasis (1–3, 13). Although excessive ROS can be damaging, regulated ROS production may activate stress-response pathways that enhance cellular resilience and longevity (5, 7, 10). However, the mechanisms that control ROS production and the processes that determine the optimal ROS levels that elicit hormetic benefits remain poorly understood.

TORC1 is a pivotal regulator of cellular growth and proliferation (14). A decrease in the availability of certain amino acids diminishes TORC1 activity, thereby extending lifespan across various organisms, including yeast (15–17). Our previous research demonstrated that *S-*adenosyl-L-homocysteine (SAH) supplementation reduces intracellular methionine (Met) levels and induces benefits associated with Met restriction (MetR), including the inhibition of TORC1 and activation of autophagy (18, 19). In mammalian cells, the sensor protein SAMTOR (*S*-adenosyl-L-methionine [SAM] sensor upstream of TORC1) has been identified as an inhibitor of TORC1. SAMTOR exerts an inhibitory effect on TORC1 signaling by interacting with GATOR1, a guanosine 5’-triphosphatase-activating protein (GAP) for RagA/B (20). Specifically, SAMTOR functions as a direct sensor of SAM. Under Met-sufficient conditions, SAM binds to the SAM-binding pocket of SAMTOR, preventing its interaction with GATOR1. Under Met-limited conditions, the lack of SAM binding allows SAMTOR to physically interact with GATOR1, thereby inhibiting TORC1 signaling (20, 21). However, as yeast lacks SAMTOR homologs, it remains unclear how SAH-induced Met depletion inhibits TORC1 and whether SAH-induced TORC1 inhibition is involved in ROS production in yeast.

In this study, we demonstrate the hormetic nature of yeast CLS by uncovering a regulatory axis governed by SAH hydrolase activity, which modulates intracellular SAH levels. We find that lifespan-extending concentrations of SAH correlate with a moderate and transient (mid-logarithmic phase) ROS accumulation. This SAH-mediated cascade induces the expression of the vacuolar adenosine 5’-monophosphate (ATP)-binding cassette (ABC) transporter gene *YBT1*, which depletes the cytosolic SAM pool and suppresses TORC1 signaling. By defining this framework, we provide a molecular resolution of how early-phase mitochondrial ROS are converted into essential signals for cellular resilience and longevity.

## Results

### SAH-Induced ROS Generation Is Necessary for Extending CLS.

**Fig. 1.**
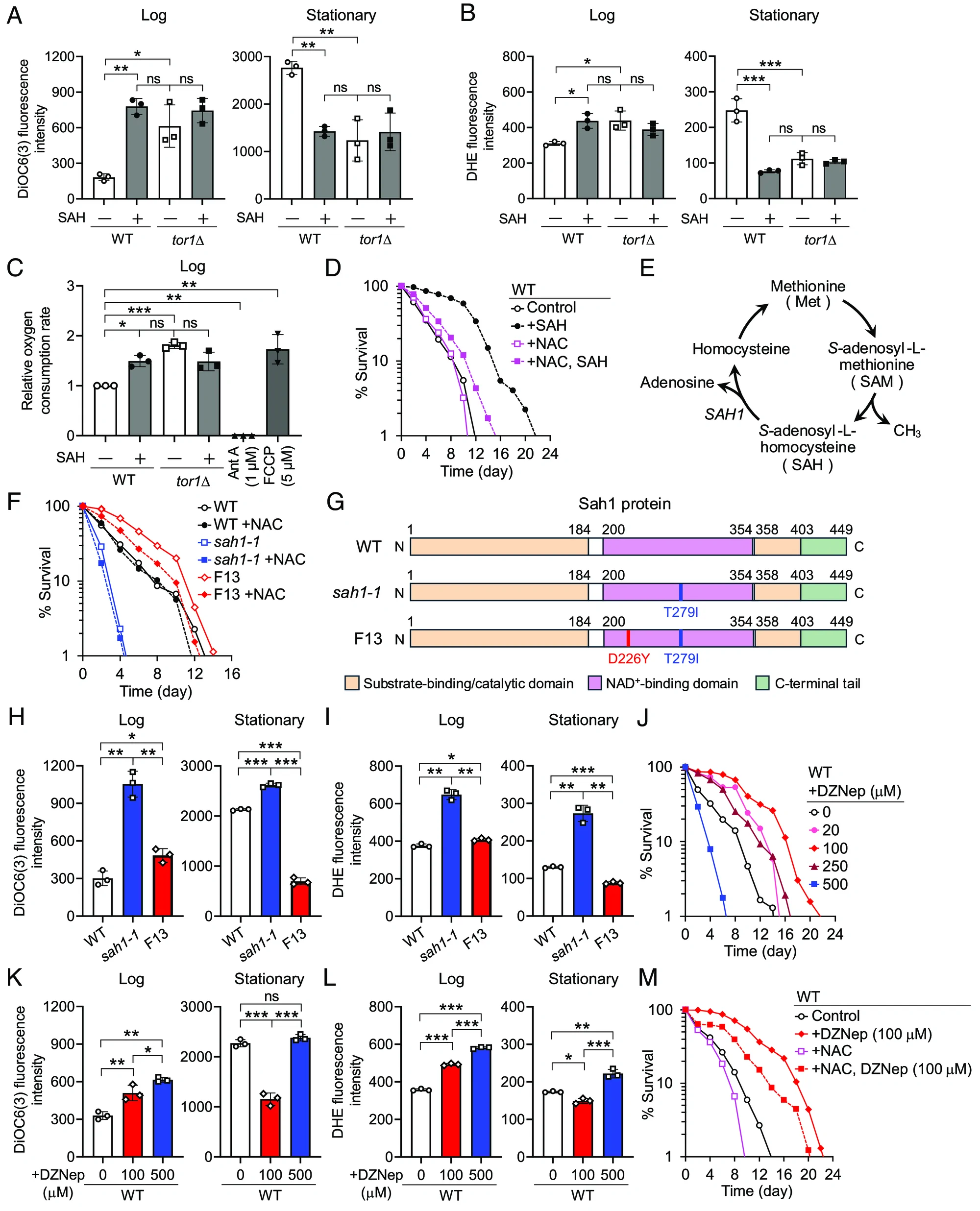
SAH supplementation or partial SAH hydrolase inhibition promotes hormesis and CLS extension. (*A and B*) Flow cytometry analysis of MMP (*A*) and ROS levels (*B*) in wild-type (WT) (BY5701; W303-1A) and *tor1*Δ (YMA32) cells cultured in SDC medium at 25 °C in the presence or absence of 1 mM SAH (*n* = 3). (*C*) Oxygen consumption rates of WT (BY5701) and *tor1*Δ (YMA32) cells in SDC medium at 25 °C. Antimycin A (Ant A; a respiratory chain complex III inhibitor) and FCCP (a mitochondrial uncoupler) were employed as negative and positive controls, respectively. (*D*) CLS curves (*n* = 3). WT (BY5701) cells were cultured in SDC medium at 28 °C. (*E*) Schematic representation of the methionine metabolic pathway in *S. cerevisiae*. Intracellular SAH levels are tightly regulated by the yeast *SAH1* gene, which encodes an SAH hydrolase. (*F*) CLS survival curves (*n* = 3). WT (BY5701), *sah1-1* (YMM222), and F13 (F13) cells were cultured in SDC medium at 28 °C. (*G*) Schematic representation of Sah1 protein structure and mutation sites, showing domain organization in WT cells and locations of the *sah1-1* (T279I) and F13 (D226Y/T279I) mutations. (*H* and *I*) Flow cytometry analysis of MMP (*H*) and ROS levels (*I*) in WT (BY5701), *sah1-1* (YMM222), and F13 (F13) cells in SDC medium at 25 °C (*n* = 3). (*J*) CLS survival curves (*n* = 3). WT (BY5701) cells were cultured in SDC medium at 28 °C. (*K* and *L*) Flow cytometry analysis of MMP (*K*) and ROS levels (*L*) in WT (BY5701) cells in SDC medium at 25 °C (*n* = 3). (*M*) CLS survival curves (*n* = 3). WT (BY5701) cells were cultured in SDC medium at 28 °C. Data are presented as mean ± SD from three biological replicates. Statistical significance was determined by two-way ANOVA (*A*–*C*) or one-way ANOVA (*H*, *I*, *K*, and *L*) with Tukey’s post hoc test, or two-way ANOVA for the survival curves (*D*, *F*, *J*, and *M*). *P < 0.05, **P < 0.01, ***P < 0.001; ns, not significant. The detailed statistical values are provided in Dataset S1.

Given our previous finding that SAH supplementation increases TCA cycle metabolites and reduces TORC1 activity (19), we anticipated a metabolic shift toward respiration. To assess mitochondrial activity and ROS production, 1 mM SAH was administered during the early logarithmic phase (OD_600_ ~ 0.1). Mitochondrial membrane potential (MMP) and ROS levels were measured using DiOC6(3) and dihydroethidium (DHE) staining, respectively (11), at the mid-logarithmic (OD_600_ ~ 1.0) and early stationary (OD_600_ ~ 4.0) phases. Because SAH-treated cells grew slower than untreated controls (19), all comparative analyses were performed at identical optical density (OD_600_) values to ensure physiological equivalence. SAH-treated wild-type (WT) cells exhibited a statistically significant increase in MMP and ROS during the mid-logarithmic phase but a decrease in the early stationary phase compared to untreated WT cells, mirroring the *tor1*Δ strains (Fig. 1 *A* and *B*). Furthermore, SAH treatment did not cause any further increase in MMP and ROS levels in *tor1*Δ cells (Fig. 1 *A* and *B*), indicating that SAH drives mitochondrial respiration through Tor1 inhibition. Consistent with these findings, analysis during the early-to-mid-logarithmic phase (OD_600_ ~ 0.2) revealed that SAH-supplemented WT cells exhibited an elevated oxygen consumption rate comparable to that of *tor1*Δ cells (Fig. 1*C*). In a *CYC3* gene deletion strain lacking cytochrome c heme lyase (12), SAH supplementation failed to increase ROS levels during the mid-logarithmic phase (*SI Appendix*, Fig. S1*A*), confirming that SAH-induced ROS production predominantly originated from the mitochondria.

Because ROS can act as signaling molecules with hormetic effects on longevity (22), we examined the impact of the ROS scavenger *N*-acetyl-cysteine (NAC) (23) on the chronological lifespan (CLS) (24). SAH extended WT CLS, consistent with previous reports (18, 19) (Fig. 1*D* and Dataset S1). Simultaneous administration of NAC abolished CLS extension (Fig. 1*D* and Dataset S1), demonstrating that log-phase ROS generation is indispensable for SAH-mediated CLS extension. Additionally, when cells pretreated with NAC were transferred to fresh medium and subsequently exposed to SAH, the CLS-extending effect of SAH was not observed (*SI Appendix*, Fig. S1*B* and Dataset S1). This demonstrates that NAC acts intracellularly to counteract the effects of SAH, rather than through direct extracellular interactions.

### Dual Nature of ROS Depends on the Degree of SAH Hydrolase Dysfunction.

The yeast *SAH1* gene, which encodes SAH hydrolase, strictly controls intracellular SAH levels (25) (Fig. 1*E*). We previously identified 15 intragenic suppressors of *sah1-1* growth retardation (18). Notably, three of these suppressors exhibited an extended CLS compared to WT cells, exemplified by the F13 strain (Fig. 1*F* and Dataset S1). Sequence analysis revealed that these long-lived mutants carried a D226Y mutation within a conserved region, occurring in tandem with the initial T279I mutation of *sah1-1* (Fig. 1*G* and *SI Appendix*, Fig. S2 *A* and *B*) (26). Although the D226Y single mutant showed a WT-like CLS, introducing D226Y into *sah1-1* recapitulated the extended CLS of F13 (*SI Appendix*, Fig. S2*C* and Dataset S1) and rescued *sah1-1* growth defects (*SI Appendix*, Fig. S2*D*), confirming D226Y as the causative mutation.

We next assessed the MMP and ROS levels in the short-lived *sah1-1* and long-lived F13 mutants. The *sah1-1* strain exhibited persistently elevated MMP and ROS levels in the early stationary phase (Fig. 1 *H* and *I*). Conversely, the F13 mutant displayed a transient log-phase ROS spike, followed by a decrease in the stationary phase (Fig. 1 *H* and *I*), mirroring the results observed in SAH-treated or *tor1*Δ cells. The extended CLS of F13 was abolished following NAC treatment (Fig. 1*F* and Dataset S1), supporting the hormetic mechanism. In contrast, various high concentrations of NAC failed to extend the CLS of *sah1-1* cells (*SI Appendix*, Fig. S2*E* and Dataset S1), suggesting that persistent and excessive ROS accumulation causes irreversible cellular damage.

Given that both mutations map to the nicotinamide adenine dinucleotide (NAD^+^)-binding domains of Sah1 (*SI Appendix*, Figs. S2 *A* and *B*), we hypothesized that the degree of SAH hydrolase activity dictates this hormetic threshold; to test this pharmacologically, we treated WT cells with the SAH hydrolase inhibitor 3-Deazaneplanocin A (DZNep) (27, 28). DZNep extended CLS in a concentration-dependent manner, peaking at 100 µM, whereas 500 µM DZNep shortened lifespan (Fig. 1*J* and Dataset S1). Crucially, 500 µM DZNep led to persistently elevated MMP and ROS levels, mimicking the *sah1-1* phenotype (Fig. 1 *K* and *L*). In contrast, 100 µM DZNep induced a transient increase in MMP and ROS during the mid-logarithmic phase, which subsided in the early stationary phase (Fig. 1 *K* and *L*), precisely mirroring the F13 mutant phenotype. Because this CLS extension by 100 µM DZNep was suppressed by NAC (Fig. 1*M* and Dataset S1), we concluded that a moderate reduction in SAH hydrolase activity elicits a beneficial hormetic ROS response.

### Partial Inhibition of SAH Hydrolase Activity Induces Benefits Mediated by MetR and Activates AMPK.

**Fig. 2.**
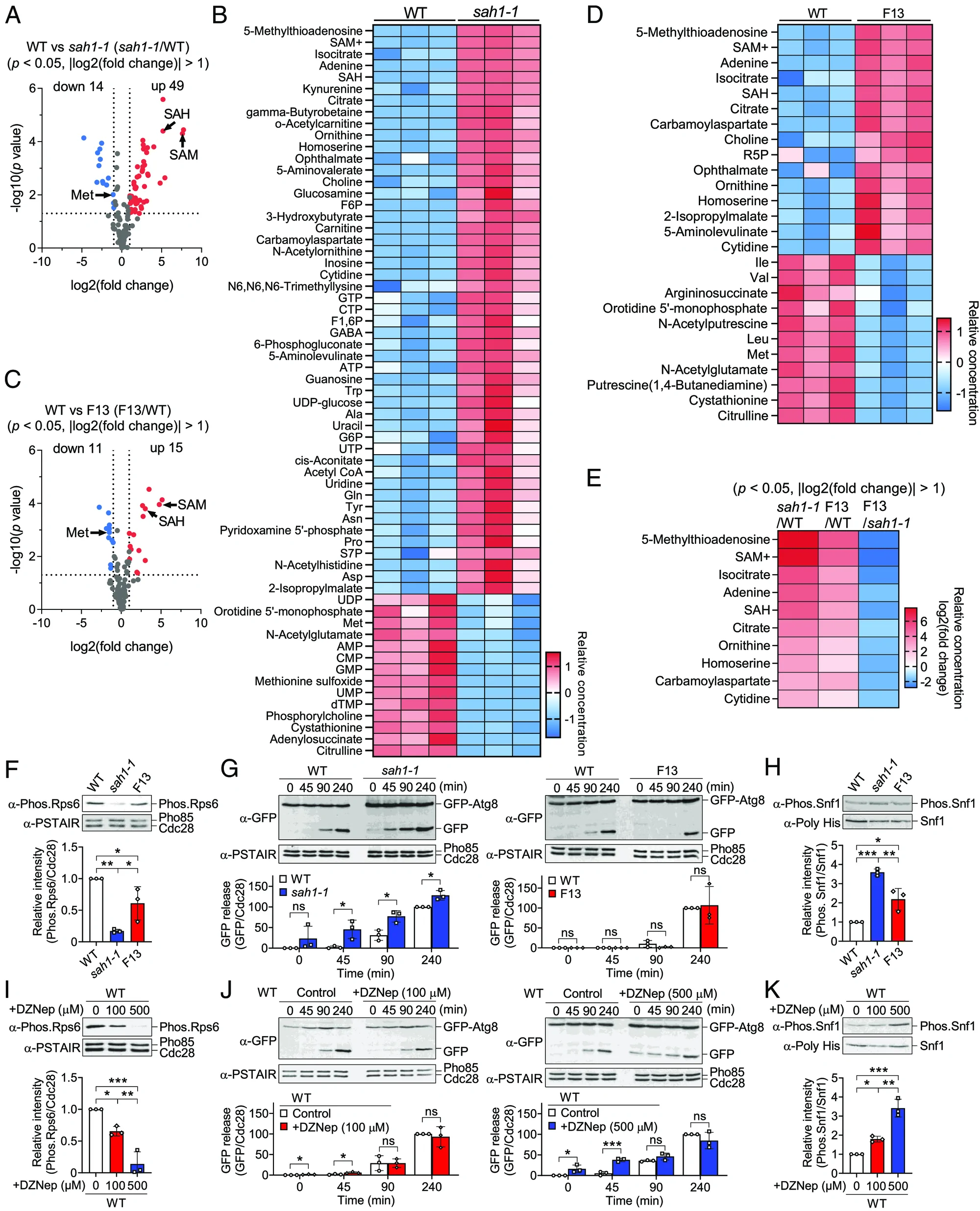
SAH hydrolase inhibition mimics a MetR state and activates AMPK. (*A*–*E*) Volcano plots (*A* and *C*) and heatmaps (*B*, *D*, and *E*) showing metabolite profiles in WT (BY5701; W303-1A), *sah1-1* (YMM222), and F13 (F13) strains (*P* < 0.05, |log2(fold change)| > 1). Detailed data are presented in Dataset S2. (*F*–*K*) Immunoblot analysis of phosphorylated Rps6 (*F* and *I*), free-GFP (*G* and *J*), and phosphorylated Snf1/AMPK (*H* and *K*), normalized to Cdc28 or total Snf1 (*n* = 3). Cells were cultured under the following conditions: (*F* and *H*) WT (BY5701), *sah1-1* (YMM222), and F13 (F13) cells in SDC medium at 25 °C; (*G*) WT (BY5701), *sah1-1* (YMM222), and F13 (F13) cells shifted from SD-Ura to SD-N medium at 25 °C; (*I* and *K*) WT (BY5701) cells in SDC medium at 25 °C; (*J*) WT (BY5701) cells were shifted from SD-Ura to SD-N medium at 25 °C. Data are presented as mean ± SD. Statistical significance was determined using one-way ANOVA with Tukey’s post hoc test (*F*, *H*, *I*, and *K*) or two-sided unpaired *t* test (*G* and *J*). *P < 0.05, **P < 0.01, ***P < 0.001; ns, not significant.

To elucidate the metabolic basis of SAH hydrolase-mediated CLS extension, we performed capillary electrophoresis time-of-flight mass spectrometry (CE-TOFMS) metabolomics on *sah1-1* and F13 mutants during the early-to-mid-logarithmic phase of growth. As anticipated, both strains exhibited significant accumulation of SAH and its precursor, SAM (Fig. 2 *A*–*D* and Dataset S2). Furthermore, certain TCA cycle metabolite levels were elevated in both mutants (Fig. 2 *B* and *D*, *SI Appendix*, Figs. S3–S6, and Dataset S2), consistent with the enhanced mitochondrial membrane potential observed previously (Fig. 1*H*). Notably, all metabolites elevated in F13 relative to WT cells, except ribose 5-phosphate (R5P), were also increased in *sah1-1* (Fig. 2 *B* and *D*, *SI Appendix*, Fig. S7, and Dataset S2). These shared metabolites accumulated at higher levels in *sah1-1* than in F13 (Fig. 2*E*), which correlated with the degree of SAH hydrolase restriction.

We previously demonstrated that SAH supplementation extends CLS by inducing a methionine restriction (MetR) state and activating adenosine 5’-monophosphate (AMP)-activated protein kinase (AMPK) (18, 19). Our metabolomic analysis revealed that Met was consistently depleted among all amino acids in both *sah1-1* and F13 mutants (Fig. 2 *B* and *D* and Dataset S2). Moreover, the metabolic profiles of *sah1-1* and F13 closely mirrored those induced by exogenous SAH supplementation (*SI Appendix*, Fig. S8 and Dataset S2), confirming that the genetic reduction of SAH hydrolase activity phenocopies SAH exposure. Therefore, we used these mutant strains to examine their effects on AMPK activation (29–31) and the benefits of MetR (32), such as reduced TORC1 activity and induction of autophagy (Fig. 2 *F*–*H*). Both *sah1-1* and F13 strains exhibited decreased phosphorylation of Rps6, a downstream target of TORC1 (33) (Fig. 2*F*), indicating TORC1 inhibition. Moreover, while the F13 mutant and WT showed no difference in baseline autophagy, the *sah1-1* strain exhibited a significant increase in vacuolar degradation of the autophagy marker GFP-Atg8, releasing free GFP (which is highly resistance to vacuolar proteases) (34) (Fig. 2*G*). Furthermore, both mutants showed increased phosphorylation of Snf1/AMPK (Fig. 2*H*), demonstrating that SAH hydrolase dysfunction promotes the activation of AMPK.

To clarify how these pathways interface with lifespan regulation, we examined TORC1, autophagy, and AMPK activities under pharmacological SAH hydrolase inhibition using DZNep at longevity-extending (100 µM) or longevity-shortening (500 µM) concentrations (Fig. 2 *I*–*K*). Interestingly, both concentrations successfully inhibited TORC1 (Fig. 2*I*). Although autophagy induction by 100 µM DZNep was subtle under standard exposure (Fig. 2*J*), an extended exposure of a replicate blot using the same samples clearly visualized a distinct increase in free GFP (*SI Appendix*, Fig. S9), confirming that both concentrations induced autophagy (Fig. 2*J*). Finally, both concentrations activated AMPK (Fig. 2*K*). Importantly, these hallmarks of lifespan extension were more pronounced under severe restriction (*sah1-1* or 500 µM DZNep) than under moderate restriction (F13 or 100 µM DZNep) (Fig. 2 *F*–*K*), despite the shortened CLS under severe conditions (Fig. 1 *F* and *J*). Collectively, these findings reinforce that partial, rather than complete, inhibition of SAH hydrolase is required to balance beneficial longevity signaling against deleterious toxicity, driving the hormetic effect on CLS.

### Overexpression of the ABC Transporter *YBT1* Elicited a Hormetic Effect on CLS.

**Fig. 3.**
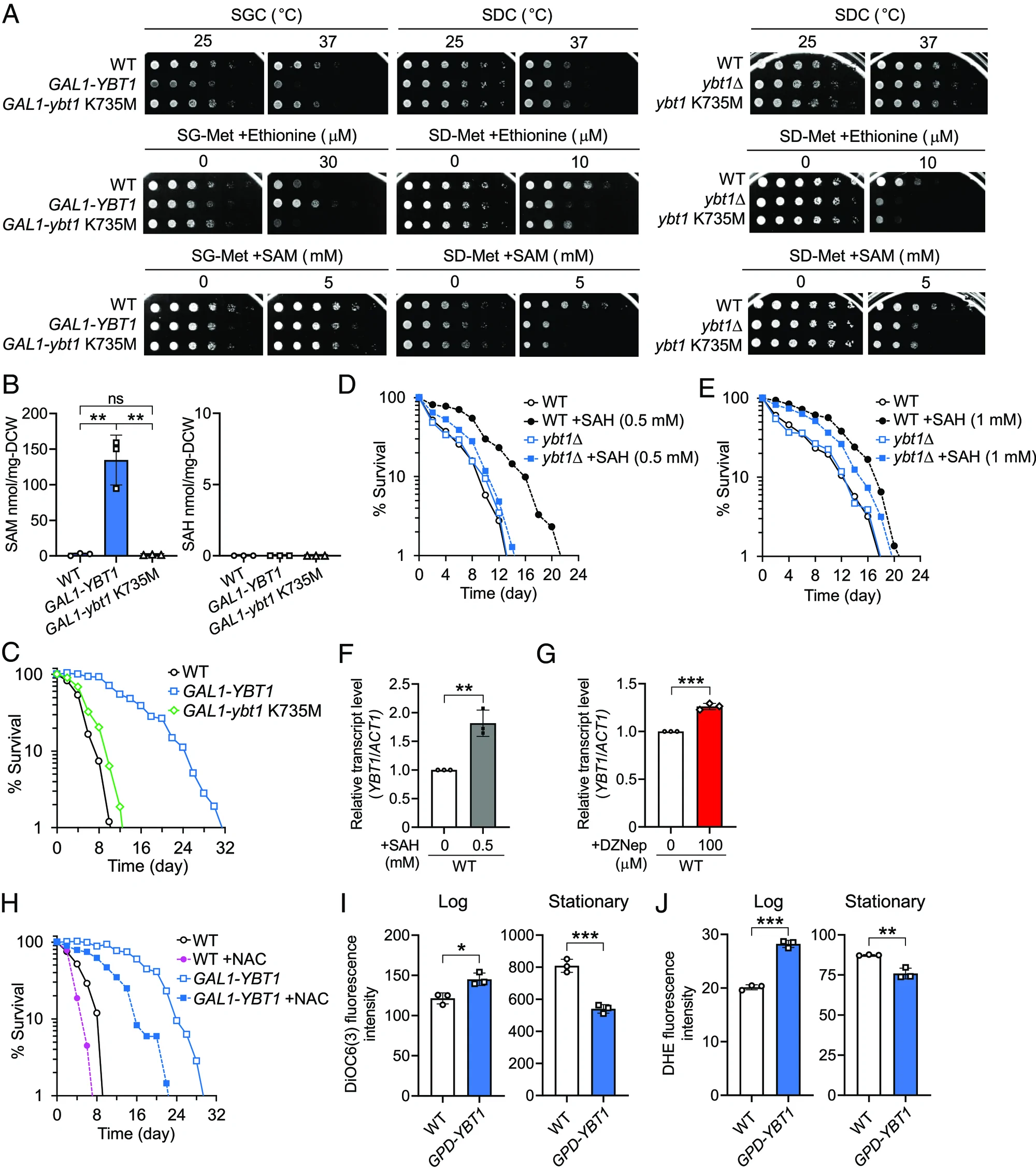
*YBT1* overexpression induces ROS-mediated hormesis to extend CLS. (*A*) Serial dilution growth assays of WT (BY5701; W303-1A), *GAL1-YBT1* (YMA145), *GAL1*-*ybt1* K735M (YMA265), *ybt1*Δ (YMA241), and *ybt1* K735M (YMA280) cells spotted on SGC, SDC, SG-Met, or SD-Met plates at 25 °C or 37 °C. Growth at 25 °C and 37 °C was compared to evaluate temperature-stress sensitivity of the strains. Separately, ethionine and SAM sensitivities were examined at 25 °C; different ethionine concentrations were optimized under SGC and SDC conditions to account for carbon source-dependent variations and to clearly distinguish the mutant phenotypes. (*B*) Intracellular SAM and SAH levels of WT (BY5701), *GAL1-YBT1* (YMA145), and *GAL1-ybt1* K735M (YMA265) cells in SGC medium at 25 °C (*n* = 3). (*C*–*E*) CLS survival curves (*n* = 3) at 28 °C. Strains and media were as follows: (*C*) WT (BY5701), *GAL1-YBT1* (YMA145), and *GAL1-ybt1* K735M (YMA265) cells in SGC medium; (*D* and *E*) WT (BY5701) and *ybt1*Δ (YMA241) cells in SDC medium. (*F* and *G*) Relative expression of *YBT1* in WT (BY5701) cells cultured in SDC medium at 25 °C (*n* = 3) treated with 0.5 mM SAH (*F*) or 100 µM DZNep (*G*), normalized to untreated controls. (*H*) CLS survival curves (*n* = 3). WT (BY5701) and *GAL1-YBT1* (YMA145) cells were cultured in SGC medium at 28 °C. (*I* and *J*) Flow cytometry analysis of MMP (*I*) and ROS levels (*J*) in WT (STY3807; X2180-1B *ura3*Δ) and *GPD-YBT1* (YMA245) cells cultured in SDC medium at 25 °C (*n* = 3). Data are presented as mean ± SD. Statistical significance was determined using one-way ANOVA with Tukey’s test (*B*), two-sided unpaired *t* test (*F*, *G*, *I*, and *J*), or two-way ANOVA for the survival curves (*C*–*E* and *H*). *P < 0.05, **P < 0.01, ***P < 0.001; ns, not significant. A detailed statistical analysis is provided in Dataset S1.

We also investigated how SAH and SAH hydrolase regulate ROS production. Although SAH supplementation increases SAM levels and extends CLS (18, 19), the transporter that moves SAM into its primary storage site, the vacuole (35), remains unknown. Consequently, guided by annotations in the Saccharomyces Genome Database (SGD), we screened available knockout strains for amino acid transporters and ATP-binding cassette (ABC) transporters specifically predicted to localize to the vacuolar membrane. Furthermore, because our previous analysis identified *ERC1*/*SSG1* as a determinant of ethionine resistance, we expanded the screen to include both multidrug and toxic compound extrusion (MATE) family genes (*ERC1/YHR032W* and *YDR338C*), despite their unknown localization to the vacuolar membrane (18, 36). This targeted screening aimed to identify transporters sensitive to ethionine, a structural analog of Met (Fig. 3*A* and *SI Appendix*, Fig. S10 *A* and *B*). Inside the cells, ethionine is converted to toxic *S*-adenosyl-L-ethionine (SAE) (*SI Appendix*, Fig. S10*A*). Thus, cells lacking a vacuolar SAM/SAE transporter would fail to sequester SAE into the vacuole, exhibiting ethionine sensitivity. Our screening revealed that only the *ybt1*Δ strain, which lacks an ATP-binding cassette class C (ABCC) transporter (37, 38), exhibited pronounced sensitivity to both ethionine and exogenous addition of high-concentration SAM (Fig. 3*A*). Conversely, galactose-induced *YBT1* overexpression conferred ethionine resistance (Fig. 3*A*). While Ybt1 has been implicated in Ni^2+^ and methotrexate transport (39, 40), overexpression of a mutated conserved ATP-binding domain (*GAL1-ybt1* K735M) (41–43) abolished resistance to ethionine, Ni^2+^, and methotrexate (Fig. 3*A* and *SI Appendix*, Fig. S10*C*), confirming that Ybt1 functions strictly through its transport activity.

Next, we investigated whether *YBT1* overexpression phenocopies the metabolic and longevity effects of SAH supplementation (18, 19). *YBT1* overexpression triggered a marked accumulation of SAM—but not SAH—and significantly extended CLS in a transporter activity-dependent manner (Fig. 3 *B* and *C* and Dataset S1). Conversely, in *ybt1*Δ strains, the longevity effect of a moderate dose of SAH (0.5 mM) was substantially diminished (Fig. 3*D* and Dataset S1), although a higher dose (1.0 mM) still extended lifespan (Fig. 3*E* and Dataset S1), suggesting both Ybt1-dependent and -independent mechanisms of SAH-mediated longevity. We further tested whether SAH directly regulates *YBT1*. Exogenous SAH (0.5 mM) or pharmacological SAH hydrolase inhibition (100 µM DZNep) significantly upregulated *YBT1* transcription (Fig. 3 *F* and *G*), suggesting that SAH promotes CLS, at least in part, by inducing *YBT1* expression, whereas additional pathways likely contribute to the full spectrum of SAH-mediated metabolic remodeling.

Finally, we explored the downstream consequences of Ybt1 activation. NAC-mediated ROS scavenging partly mitigated CLS extension in *YBT1*-overexpressing cells (Fig. 3*H* and Dataset S1), indicating a hormetic response. To monitor mitochondrial dynamics, we initially evaluated two distinct genetic backgrounds (*SI Appendix*, Fig. S11). Specifically, when *YBT1* was overexpressed via the GPD (glyceraldehyde-3-phosphate dehydrogenase) promoter in glucose medium, the W303 background eventually achieved CLS extension (*SI Appendix*, Fig. S11*A* and Dataset S1). However, its initial survival declined rapidly, similar to that of the WT control, rendering this background unsuitable for tracking early mitochondrial functions. Therefore, the X2180 strain, which successfully preserved the initial survival under these conditions (*SI Appendix*, Fig. S11*B* and Dataset S1), was used to investigate mitochondrial dynamics. Consistent with SAH supplementation, *GPD-YBT1* in the X2180 background induced a transient elevation in both MMP and ROS levels during the mid-logarithmic phase (OD_600_ ~ 1.0), which subsequently subsided in the early stationary phase (OD_600_ ~ 7.0) (Fig. 3 *I* and *J*). Collectively, these results reinforce that SAH drives longevity, at least in part, by inducing Ybt1-mediated vacuolar SAM sequestration and a subsequent beneficial hormetic ROS response.

### Overexpression of *YBT1* Enhances Vacuolar Sequestration of SAM.

**Fig. 4.**
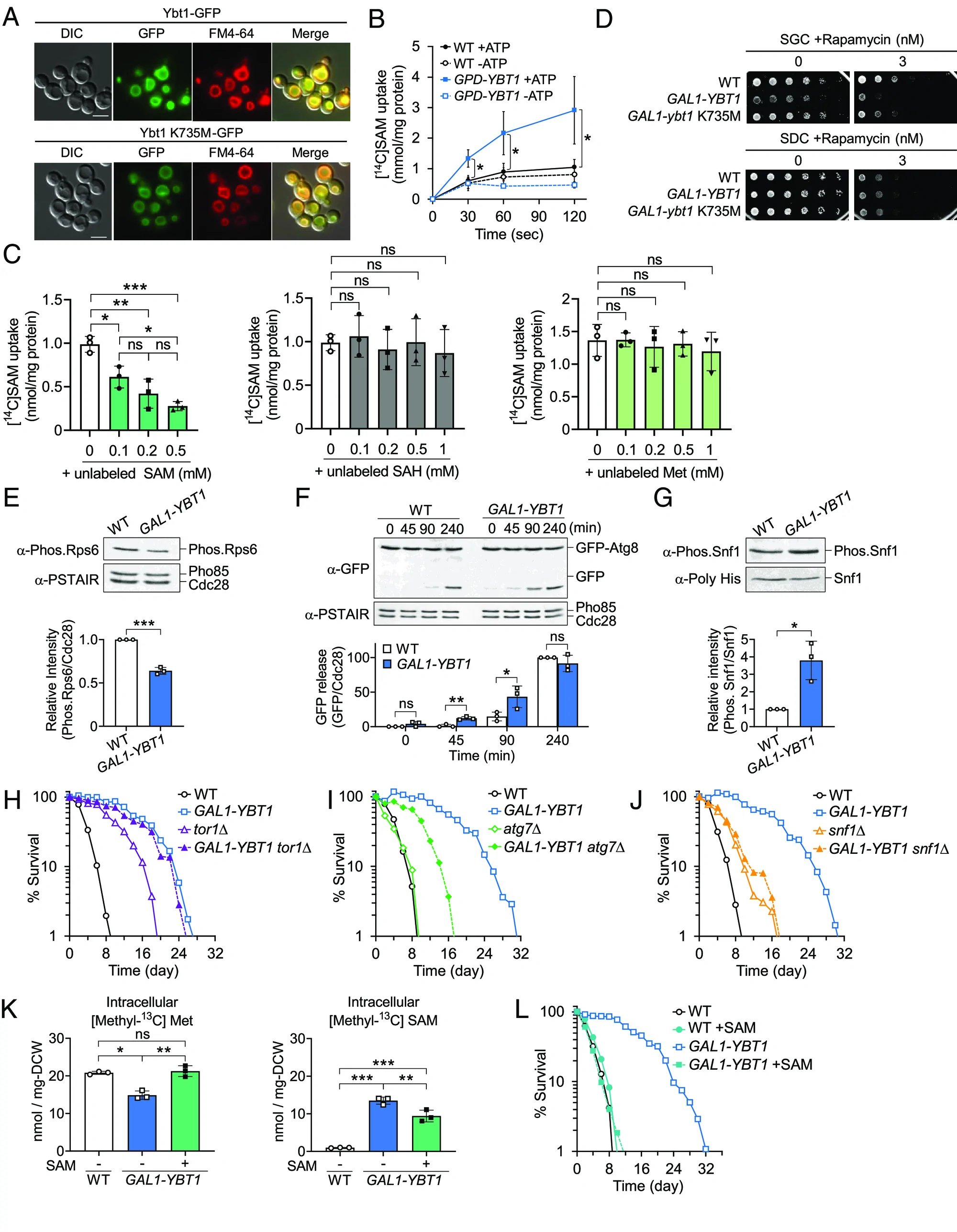
Ybt1 mediates vacuolar SAM transport to modulate TORC1, autophagy, and AMPK activities. (*A*) Fluorescence microscopy of Ybt1-GFP (YMA176) and mutant Ybt1(K735M)-GFP (YMA228) cells cultured in SDC medium at 25 °C, costained with FM4-64 to verify vacuolar localization. (Scale bar, 5 µm.) (*B* and *C*) [^14^C]SAM transport activity (*B*) and competitive inhibition (*C*) using isolated vacuolar membrane vesicles from cells cultured in YPD medium at 30 °C. The transport activity assays using the isolated vacuolar membrane vesicles were performed at 25 °C. (*B*) Time-dependent [^14^C]SAM uptake into WT (STY3807; X2180-1B *ura3*Δ) and *GPD-YBT1* (YMA245) vesicles, demonstrating ATP-dependent transport. (*C*) Competition assays using *GPD-YBT1* vesicles, revealing Ybt1 substrate specificity. (*D*) Serial dilution growth assay of WT (BY5701; W303-1A), *GAL1-YBT1* (YMA145), *GAL1-ybt1* K735M (YMA265) cells spotted on SGC or SDC plates at 25 °C to evaluate mutant phenotypes. (*E*–*G*) Immunoblot analysis of phosphorylated Rps6 (*E*), free-GFP (*F*), and phosphorylated Snf1/AMPK (*G*) in WT (BY5701) and *GAL1-YBT1* (YMA145) cells (*n* = 3) to monitor signaling activities, with cells expressing GFP-Atg8 shifted from SG-Ura to SG-N medium in (*F*). (*H*–*J*) CLS survival curves (*n* = 3) in SGC medium at 28 °C. WT (BY5701) and *GAL1-YBT1* (YMA145) cells are included in all panels, with the following additional strains: (*H*) *tor1*Δ (YMA32) and *GAL1-YBT1 tor1*Δ (YMA276); (*I*) *atg7*Δ (YYK1) and *GAL1-YBT1 atg7*Δ (YMA268); and (*J*) *snf1*Δ (YTK22) and *GAL1-YBT1 snf1*Δ (YMA270). (*K*) CE-TOFMS analysis of intracellular [methyl-^13^C]Met and [methyl-^13^C]SAM levels in WT (BY5701) and *GAL1-YBT1* (YMA145) cells in SR medium at 25 °C. Cells were pretreated with water vehicle (WT and *GAL1-YBT1*) or 1 mM SAM (*GAL1-YBT1*) for 1 h, followed by 6 h of 2% galactose induction for the tracer analysis. (*L*) CLS survival curves (*n* = 3). WT (BY5701) and *GAL1-YBT1* (YMA145) cells were cultured in SGC medium at 28 °C. Data are presented as mean ± SD. Statistical significance was determined by the two-sided unpaired *t* test (*B* and *E*–*G*), one-way ANOVA with Tukey’s test (*C* and *K*), or two-way ANOVA for the survival curves (*H*–*J* and *L*). *P < 0.05, **P < 0.01, ***P < 0.001; ns, not significant. Detailed statistics are provided in Dataset S1.

To delineate the role of Ybt1, we confirmed that both Ybt1-GFP and mutated Ybt1 (K735M)-GFP colocalized with the vacuolar membrane dye FM4-64 (44) (Fig. 4*A*). We then assessed whether Ybt1 functions as a SAM transporter. Using vacuolar membrane vesicles purified from *GPD-YBT1*-overexpressing X2180 cells, in vitro assays revealed robust ATP-dependent [^14^C]SAM uptake (Fig. 4*B*). Furthermore, competition assays demonstrated that the accumulation of [^14^C]SAM uptake was effectively abolished by the addition of unlabeled SAM, whereas neither SAH nor Met exerted such an inhibitory effect (Fig. 4*C*). These data establish that Ybt1 functions as a vacuolar SAM transporter with high substrate specificity.

### *YBT1* Overexpression Recapitulates the Longevity Benefits of MetR and AMPK Activation.

As *YBT1* overexpression mimics the effects of SAH (18, 19), we hypothesized that it engages a similar metabolic signaling network. To test whether *YBT1* overexpression inhibits TORC1, we examined hypersensitivity to the TORC1 inhibitor rapamycin. Consistent with this notion, *YBT1*-overexpressing strains demonstrated marked sensitivity to rapamycin (Fig. 4*D*), and a substantial reduction in Rps6 phosphorylation (Fig. 4*E*), which is a definitive readout of TORC1 inactivation. Furthermore, *YBT1* overexpression enhanced the degradation of GFP-Atg8 into free GFP (Fig. 4*F*) and increased Snf1/AMPK phosphorylation (Fig. 4*G*), indicating autophagy and AMPK activation. When *TOR1* was disrupted in the strain overexpressing *YBT1*, no further extension of CLS was observed (Fig. 4*H*), indicating that Ybt1-mediated longevity operates, at least in part, through TORC1 inhibition. Moreover, CLS extension was completely negated in *snf1*Δ and partly in *atg7*Δ (deletion mutant of an essential autophagic machinery component) mutants (Fig. 4 *I* and *J*), suggesting that *YBT1* overexpression extends CLS, at least to some extent, through a mechanism dependent on the AMPK and autophagy pathways.

Given that SAH promotes Met consumption for SAM synthesis (18, 19), we performed stable isotope tracing with L-[methyl-^13^C]Met following *YBT1* overexpression. *YBT1*-overexpressing cells exhibited a marked decrease in intracellular [methyl-^13^C]Met levels, along with a concomitant increase in [methyl-^13^C]SAM levels (Fig. 4*K*), confirming that Ybt1 activation accelerates endogenous Met-to-SAM conversion.

**Fig. 5.**
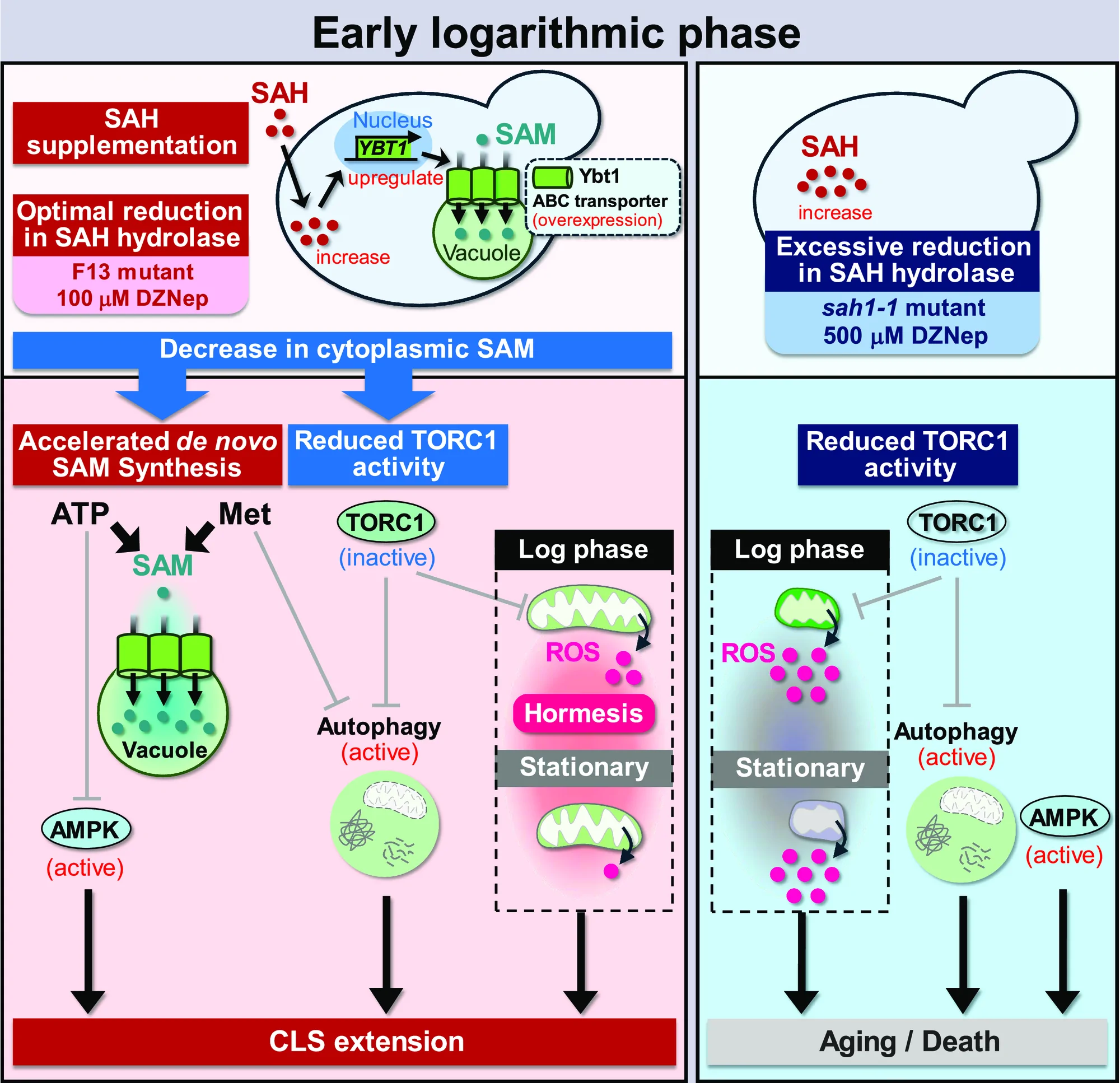
Proposed model for CLS extension via the SAH-Ybt1-TORC1 adaptive/hormetic response. SAH supplementation during the early logarithmic phase enhances mitochondrial respiration via TORC1 inhibition, generating ROS levels that are essential for the adaptive/hormetic response. We propose that SAH upregulates *YBT1* expression and activity, facilitating SAM transport into the vacuoles. The resulting decrease in cytoplasmic SAM potentially induces both a reduction in TORC1 activity and an acceleration of de novo SAM synthesis, the latter of which consumes Met and ATP to activate autophagy and AMPK. CLS is governed by a hormetic balance between pro-longevity signaling and ROS-mediated toxicity, which is regulated by SAH hydrolase. Arrows and T-bars indicate activation and inhibition, respectively.

Because vacuolar SAM sequestration reduces cytosolic SAM availability, we reasoned that this depletion promotes longevity. Supporting this, exogenous SAM supplementation suppressed CLS extension (Fig. 4*L*) and restored the decreased Met levels induced by *YBT1* overexpression (Fig. 4*K*). These findings suggest that Ybt1-mediated vacuolar SAM transport curtails cytosolic SAM pools, thereby driving longevity through a hormetic mechanism (Fig. 5).

## Discussion

The dualistic nature of ROS has long positioned them at the intersection of cellular damage and cell survival. While hormesis is well established in model organisms (2–8), the molecular transducers that convert metabolic shifts into beneficial ROS signals remain unknown. Our findings resolve this paradox by identifying a direct regulatory axis in which SAH-mediated TORC1 suppression is coupled with ABC transporter-driven cascade that elicits hormetic ROS signals to promote CLS (Fig. 5).

Specifically, SAH drives a shift in mitochondrial respiration by inhibiting TORC1, leading to a transient elevation in MMP and ROS levels during the mid-logarithmic phase, followed by their maintenance at low levels in the early stationary phase. As reported previously (11), this phenomenon functions as a conserved adaptive mechanism that extends CLS. Importantly, the activity of SAH hydrolase determines this hormetic window in vivo: moderate inhibition (100 µM DZNep or F13 mutant) activates pro-longevity signals such as autophagy, AMPK, and TORC1 inhibition without exceeding toxicity thresholds, whereas excessive inhibition (500 µM DZNep or *sah1-1* mutant) triggers deleterious ROS accumulation (Figs. 1 *I* and *L* and 2 *F*–*K*). This concentration-dependent balance highlights the delicate metabolic equilibrium required for mitochondria-mediated CLS extension in response to SAH. By coordinating mitochondrial quality control and biogenesis, these SAH-induced pathways likely function in an integrated manner to maintain mitochondrial homeostasis, thereby facilitating this mitochondria-mediated longevity (31, 45–49).

Consistent with our yeast observations, nematode SAH hydrolase mutants exhibit extended lifespans (50), and exogenous SAH extends the lifespan of wild-type worms in a concentration-dependent manner (19). Furthermore, a cohort study revealed that centenarians in Spain exhibit increased plasma SAH levels compared to younger adults and the elderly (51). Although these human data remain correlational, they imply that SAH levels are evolutionarily associated with age-related biological processes in diverse species.

A pivotal effector in this SAH-induced longevity pathway is the ABC transporter, Ybt1. Both SAH supplementation and SAH hydrolase inhibition transcriptionally activated *YBT1* (Fig. 3 *F* and *G*), and its overexpression was sufficient to extend CLS via ROS-mediated hormesis (Fig. 5). This mechanism relies on spatial SAM redistribution, wherein Ybt1 sequesters SAM into the vacuole, thereby depleting the cytosolic SAM pool. While mammals utilize the nutrient sensor SAMTOR to couple cytosolic SAM availability directly to TORC1, yeast lacks a strict SAMTOR homolog.

Instead, we propose a molecular model in which *YBT1* overexpression depletes the cytosolic SAM pool, thereby impairing the SAM-dependent methyltransferase, Ppm1. Ppm1-mediated methylation of PP2A normally sustains active TORC1 signaling (52). In this scenario, the restriction of this methyltransferase pathway could suppress the downstream TORC1-Sch9 axis, leading to the observed downregulation of Rps6 phosphorylation (Fig. 4*E*). This physical partitioning reconciles the apparent paradox of elevated total cellular SAM levels without a concomitant increase in SAH (Fig. 3*B*), as vacuolar sequestration effectively limits the availability of substrates for cytosolic methyltransferases. While exogenous SAM supplementation counteracts the pro-longevity effects of *YBT1* overexpression, the complex interplay between cytosolic SAM dynamics and the threshold of TORC1 reactivation remains to be fully elucidated, potentially governed by specific nutrient or media conditions. Ultimately, this optimized cytosolic methylation state suppresses pro-aging programs while preserving essential cellular homeostasis.

Additionally, yeast supplementation with SAH (19) or *YBT1* overexpression promotes Met consumption, resulting in accelerated conversion to SAM (Fig. 5). This accelerated synthesis suggests the activation of a compensatory feedback loop to replenish the cytosolic SAM pool, which is continuously depleted via Ybt1-mediated vacuolar sequestration. These findings suggest that Ybt1 is involved not only in SAM transport to the vacuole but also in SAM synthesis. Consequently, this persistent demand for SAM synthesis drives a marked decrease in intracellular Met levels, effectively phenocopying a MetR state.

MetR is known to extend lifespan and delay aging-associated pathologies in most model organisms (32, 53). Although our finding that SAH induces an early transient ROS burst appears to contradict the conventional view that MetR reduces mitochondrial ROS (54), these outcomes are complementary rather than contradictory. While that review highlighted the chronic, steady-state reduction in ROS observed after prolonged MetR, our data focused on the early growth phase. We demonstrated that a transient ROS signal at this early stage is required as a primary trigger to activate downstream antiaging programs, which ultimately leads to a low-ROS state during the subsequent stationary phase, as evidenced by our NAC rescue experiments (Figs. 1 *D* and *F* and 3*H*).

Beyond its implications to aging, this pathway holds significant therapeutic potential in oncology. MetR conventionally reduces cytosolic SAM levels, offering a potent strategy to suppress tumor proliferation (55, 56). Mechanistically, we demonstrated that SAH signaling and *YBT1* overexpression trigger AMPK activation via an elevated ADP/ATP ratio, driven by the increased ATP demand required for both SAM biosynthesis and vacuolar transport (18, 19, 57–60). This metabolic rewiring, coupled with the known vulnerabilities of the Warburg effect (61), highlights the SAH-Ybt1-SAM axis as a powerful target for disrupting cancer metabolism, effectively complementing existing dietary MetR strategies. Furthermore, this cellular energetic stress likely intersects with NAD^+^ metabolism to reinforce longevity. AMPK activation stimulates NAD^+^ biosynthesis, enhancing downstream sirtuin signaling (62). Additionally, because SAH hydrolases (such as yeast Sah1) are evolutionarily conserved NAD^+^-dependent enzymes that rely on bound NAD^+^ for their catalytic cycles (63) (Fig. 1*G*), cellular NAD^+^/NADH ratio fluctuations driven by nutritional shifts or altered mitochondrial respiration may act as an upstream rheostat for Sah1 activity. Under conditions that alter cellular redox or energetic status, subsequent changes in Sah1 activity could fine-tune the SAH hormetic window.

In conclusion, targeting the SAH-driven pathway offers promising therapeutic strategies for addressing age-related disorders and, ultimately, enhancing the human healthspan.

## Materials and Methods

All *S. cerevisiae* strains used in this study were derivatives of W303-1A, X2180-1B, and BY4742 backgrounds, with genetic modifications performed via PCR-mediated gene disruption and CRISPR/Cas9 systems. Phenotypic and physiological characterizations were conducted using chronological lifespan (CLS) assays, cell density and oxygen consumption measurements, microscopy, and flow cytometry to measure mitochondrial membrane potential and ROS. Intracellular metabolites, including SAM, SAH, and ^13^C-labeled variants, were analyzed using mass spectrometry-based metabolomics, and ATP-dependent [^14^C]SAM uptake assays were performed using isolated vacuolar membrane vesicles. Cellular signaling pathways were monitored using GFP-Atg8 processing, Rps6 activation, Snf1/AMPK activation assays, and quantitative RT-PCR. Statistical analyses, including sample size and significance calculation (*P* < 0.05), were executed using GraphPad Prism 9.0. Detailed materials and methods, including experimental procedures, tables for strains, media, and primers, are described in the *SI Appendix*.

## Supplementary Material

Appendix 01 (PDF)

Dataset S01 (XLSX)

Dataset S02 (XLSX)

Dataset S03 (XLSX)

## Data Availability

The original metabolome data have been deposited in the Metabolomics Workbench (64) under the accession code no. PR002538 (10.21228/M8KK01). All other data are included in the manuscript and/or the supporting information.
