# Supplementary material for: S-adenosyl-L-homocysteine triggers an adaptive response that extends lifespan through the YBT1 ABC transporter
Source: Proc Natl Acad Sci U S A. 2026 Oct 16;123(42):e2621286123. doi: 10.1073/pnas.2621286123 (PMC13684291; doi:10.1073/pnas.2621286123)
Supplement: Supplementary file 1 [file pnas.2621286123.sapp.pdf]

PNAS

Supporting Information for

S-adenosyl-L-homocysteine triggers an adaptive response that extends lifespan through the *YBT1* ABC transporter

Koji Masumura[1], Sayaka Kawasaki[1], Mayu Tsutsumi[1], Hiiro Yamashita[1], Muneyoshi Kanai[2], Haruto Nakajo[3], Wakaba Yagi[3], Shoma Katsunoi[3], Kaori Yoneyama[4], Miyuki Kawano-Kawada[3,5,6], Takayuki Sekito[3,5], Takafumi Ogawa[1], Tomoyoshi Soga[7,8], and Masaki Mizunuma[1,9,*]

[1]Unit of Biotechnology, Graduate School of Integrated Sciences for Life, Hiroshima University, Higashi-Hiroshima, Japan.

[2]National Research Institute of Brewing, Higashi-Hiroshima, Japan.

[3]Laboratory of Molecular Physiology and Genetics, Faculty of Agriculture, Graduate School of Agriculture, Ehime University, Matsuyama, Japan.

[4]Research and Development Bureau, Saitama University, Saitama, Japan.

[5]Division of Cell-Free Sciences, Proteo-Science Center (PROS), Ehime University, Matsuyama, Japan.

[6]Advanced Research Support Center (ADRES), Ehime University, Matsuyama, Japan

[7]Institute for Advanced Biosciences, Keio University, Tsuruoka, Japan.

[8]Human Biology-Microbiome-Quantum Research Center (WPI-Bio2Q), Keio University, Tokyo, Japan.

[9]Hiroshima Research Center for Healthy Aging (HiHA), Hiroshima University, Higashi-Hiroshima, Japan.

*Correspondence author: Masaki Mizunuma

Email: mmizu49120@hiroshima-u.ac.jp

This PDF file includes:

Materials and methods
Table S1 to S3
SI References
Figures S1 to S11

Other supporting materials for this manuscript include (separate files):
1. Datasets S1 to S3, which are available online and contain:
1.1. Dataset S1. Excel file contains detailed statistical results for the chronological lifespan (CLS) assays, including *P* values, mean lifespan, and 10% survival values.
1.2. Dataset S2. Excel file contains processed metabolomics data underlying Figs. 2A–E and SI Appendix, Figs. S3 to S8.
1.3. Dataset S3. Excel file contains the complete normalized and unfiltered metabolomics dataset.

Materials and methods

Yeast strains and media

The yeast strains used in this study are listed in Table S1. The yeast *S. cerevisiae* strains were derivatives of W303-1A, X2180-1B, and BY4742.

Gene disruption was performed using a standard PCR-based method (1). The detailed growth media, chemical compositions, and corresponding figures used in this study are summarized in Table S2.

Experimental temperature settings

While *S. cerevisiae* is conventionally cultured at 30°C, the majority of assays in this study were conducted at 25°C. This temperature was selected based on the growth properties of *sah1-1* mutant. Although the mutant exhibited slow growth even at 25°C, this growth delay became more pronounced at 30°C due to its temperature-sensitive nature. Performing the assays at 25°C minimized potential confounding variables associated with increased growth retardation and thermal stress, thereby enabling a reliable and stable comparison between the strains. Although the wild-type (WT) strain also grew slightly slower at 25°C than at 30°C, this temperature ensured the physiological stability of both strains for a fair comparison. To maintain consistency, assays that did not directly utilize the *sah1-1* strain were also performed at 25°C.

The chronological lifespan (CLS) assay was performed at 28°C, following the established standard protocol used in our laboratory to ensure direct comparability with previously accumulated baseline CLS data. Under these conditions, the CLS profile of the WT strain (W303 background) was highly comparable to previously reported data obtained at 30°C. At 28°C, the *sah1-1* mutant strain grew stably without exhibiting abnormal colony morphology or phenotypic integrity issues, thereby validating the accuracy and physiological relevance of survival rate measurements.

An *in vitro* SAM transport assay was conducted using isolated vacuolar membrane vesicles. Yeast cells were cultured at 30°C to optimize the yield and structural integrity during vesicle isolation, and the subsequent transport activity assay was performed at 25°C, following established protocols (2, 3). Conducting the transport activity assay at 25°C moderated the uptake rate compared to 30°C, thereby facilitating the accurate determination of the initial velocity. This setting also ensures methodological consistency with our other primary physiological assays.

Yeast gene disruption

The *tor1*Δ *a*nd *ybt1*Δ strains were constructed by gene replacement. Genomic DNA was isolated from *tor1*Δ*::kanMX4* and *ybt1*Δ*::kanMX4* strains with a BY4741 background (Invitrogen). The *TOR1* and *YBT1* loci were amplified by PCR using primers listed in Table S3. The resulting PCR products were used to transform the W303-1A strain.

The *cyc3*Δ strain was constructed by PCR amplification using the pFA6a-kanMX6 plasmid as a template, replacing the *CYC3* ORF in the W303-1A strain with the kanMX6 cassette using the primers listed in Table S3.

Similarly, the *rtc2*Δ strain was constructed by PCR amplification using pFA6a-kanMX6 as a template, replacing the *RTC2* ORF in the BY4742 strain with kanMX6 using the primers listed in Table S3.

Overexpression strains were constructed by chromosomal integration of expression cassettes driven by the *GAL1* or *GPD* promoter. PCR amplification was performed using plasmids pFA6a-kanMX6-PGAL1 or pYM-N14 as templates with primers listed in Table S3, and the amplified fragments were used to transform either the W303-1A or STY3807 (4) strains.

The *YBT1-GFP* strain was constructed by PCR amplification of the expression cassette containing GFP and kanMX6 using the plasmid pFA6a-GFP-kanMX6 as a template and integrated into the genome to fuse GFP to the C-terminus of the *YBT1* ORF, employing primers listed in Table S3.

Construction of CRISPR plasmids and yeast transformation

The plasmid used in this study was pGAL1-Cas9-tADH1-pGAL1-2BsaI-sgRNAFE(empty)-HDV-tCYC1-CU (5), which was obtained from the National BioResource Project (Yeast) (Japan). To design single guide RNAs (sgRNAs) for SpCas9, target sequences located upstream of the PAM site were selected using CRISPRdirect (6): 5'-GGTTACGGTGATGTCGGTAA-3' for the *SAH1* gene and 5'-TGGTAAGACATCCCTACTAA-3' for the *YBT1* gene. Corresponding single-stranded oligonucleotides (Invitrogen) were synthesized for each target genes. To incorporate 4-nt sequences compatible with the backbone vector and hammerhead ribozyme in the oligonucleotides, we employed a program developed by Okada et al. (2021) (5) (https://github.com/poccopen/Genome_editing_plasmid_for_budding_yeast), which automatically designs these sequences. The sgRNA sequence was inserted into the *Bsa* I site of the plasmid using the NEB Golden Gate Assembly Kit (*Bsa*I-HF v2) (New England Biolabs, E1601), resulting in a plasmid targeting either the *SAH1* or *YBT1* gene.

Yeast transformation for genome editing was conducted using the lithium acetate method, as described by Okada et al. (2021) (5). A single amino acid substitution in Sah1 or Ybt1 was introduced using the CRISPR-Cas9 system, as described by Okada et al. (2021) (5). The donor fragment for *SAH1* or *YBT1* was amplified using the primers listed in Table S3 with KOD One PCR Master Mix (Toyobo, KMM-101) in accordance with the manufacturer's instructions.

Dilution spot assay

Dilution spot assays were performed to assess cell growth under various conditions. Cells were cultured overnight at 25 °C in a liquid medium corresponding to the solid medium used for the assay without the test compound. Harvested cells were resuspended in sterile water at 5×10^7 cells/mL and subjected to five consecutive five-fold serial dilutions to yield six cell densities. Each dilution was spotted onto the indicated solid media using a metal pin replicator.

For temperature sensitivity assays, cells were spotted onto YPD, SDC, or SGC plates and incubated at 25 or 37 °C. For chemical sensitivity assays, cells were spotted onto SD-Met or SG-Met plates containing 10 or 30 µM ethionine (Fujifilm Wako Pure Chemical Corporation, 057-00331), or 5 mM SAM (Sigma-Aldrich, A7007). Alternatively, cells were spotted onto SDC or SGC plates containing 3 nM rapamycin (LC Laboratories, R-5000), 0.4 mM $NiCl_2$ (Fujifilm Wako Pure Chemical Corporation, 141-01045), or 0.1 mM methotrexate (MTX; Fujifilm Wako Pure Chemical Corporation, 139-13571) in 1% dimethyl sulfoxide (DMSO; Sigma-Aldrich, 07-4860-5). Plates without the corresponding compounds were used as control. Glucose- and galactose-containing plates were incubated for 2 and 3 days, respectively, before visualization.

Chronological Lifespan (CLS) assay for yeast

CLS analysis was performed using synthetic dextrose complete (SDC) or synthetic galactose complete (SGC) liquid medium, as previously described (7-9). Briefly, overnight cultures grown in SDC or SGC were diluted (2×10^6 cells/ml) in fresh SDC or SGC medium. SAH (Sigma-Aldrich, A9384) or SAM (Sigma-Aldrich, A7007) was added to achieve a final concentration of 1 mM, DZNep (Selleck, S7120) was added to a final concentration ranging from 20 to 500 µM, and NAC (Sigma-Aldrich, A7250) was added to a final concentration of 10 mM. If alternative concentrations were employed, the specific concentration is denoted in the figure. For the NAC pre-treatment experiment, as shown in Fig. S1B, overnight cultures grown in SDC were diluted (2×10^6 cells/ml) in fresh SDC medium and incubated with 10 mM NAC for 2 hours. Following incubation, the cells were washed twice with fresh SDC medium to remove residual NAC, resuspended in fresh SDC medium, and subsequently treated with SAH at a final concentration of 1 mM.

The cultures were then incubated at 28°C with shaking at 180 rpm. Cell viability was assessed by plating aging cells onto yeast extract (Oriental Yeast Co., Ltd., 42010000), peptone (Labology Co., Ltd., 399-02267), and dextrose (Nacalai Tesque, 16806-54) (YPD), 400 mg/l adenine (Sigma-Aldrich, A9126), and 200 mg/l uracil (Sigma-Aldrich, U0750) agar plates and monitoring colony-forming units (CFUs) starting from day three, which was considered the initial survival (100%). Cultures were routinely monitored until survival reached approximately 1%, except for the biological replication trial for Fig. S1B, which was prospectively terminated at a 10% survival threshold (8). All data are represented as the average of three biological replicates. At least two sets of CLS experiments were conducted, yielding similar outcomes.

CLS assays were conducted using SDC or SGC medium [0.17% yeast nitrogen base without amino acids and ammonium sulfate (Difco, 233520); 0.5% ammonium sulfate (Sigma-Aldrich, 01-5110-5); 2% glucose (Nacalai Tesque, 16806-54); amino acids to a final concentration of 20 mg/l (adenine (Sigma-Aldrich, A9126), arginine (Kanto Chemical, 01434-30), histidine (Sigma-Aldrich, 13-1160-2), methionine (Nacalai Tesque, 21719-02), tryptophan (Kanto Chemical, 40339-30), and uracil (Sigma-Aldrich, U0750)), 30 mg/l (isoleucine (Sigma-Aldrich, 15-2120-2), leucine (Sigma-Aldrich, 18-1010-2), lysine (Sigma-Aldrich, 18-1900-2), and tyrosine (Sigma-Aldrich, 30-5530-2)), 60 mg/l (phenylalanine (Kanto Chemical, 32100-30)), and 150 mg/l (valine (Sigma-Aldrich, 33-0090-2))]. The SGC medium had the same composition as the SDC medium but contained 2% (w/v) galactose (Nacalai Tesque, 16511-75) instead of glucose. GraphPad Prism software 9.0 (GraphPad Software, San Diego, CA, USA) was used to compare CLS, and *p*-values were obtained from a two-way ANOVA with time and strain used as independent factors. Detailed statistical results of the CLS assays are provided in Dataset S1.

Measurement of cell density

Cell growth was monitored by measuring the optical density at OD_{600} using a spectrophotometer (U-2900; Hitachi High-Tech Corporation, Tokyo, Japan). The linear range of this instrument was up to an OD_{600} of approximately 0.8. For cell cultures exceeding an OD_{600} of 0.4, the samples were appropriately diluted with fresh medium to obtain accurate readings within the linear range. Cell densities were quantified using a Sysmex cell counter (CDA-1000; Sysmex Corporation, Kobe, Japan).

In synthetic media, across all tested strains and treatment conditions, the cell concentrations corresponding to representative OD_{600} values for the W303 strain were as follows: 1.5–1.8 × 10^6 cells/mL at an OD_{600} of ~0.1; 3–3.6 × 10^6 cells/mL at an OD_{600} of ~0.2; 1.5–1.8 × 10^7 cells/mL at an OD_{600} of ~1.0; and 3.5–5 × 10^7 cells/mL at an OD_{600} of ~4.0. For the X2180 strain, the cell concentrations were 2.0 × 10^7 cells/mL at the mid-logarithmic phase (OD_{600} ~1.0) and 1.5 × 10^8 cells/mL at the early stationary phase (OD_{600} ~7.0) (reflecting morphological and physiological changes in high-density cultures).

Flow Cytometry for measurements of mitochondrial membrane potential (MMP) and ROS

Cells were initially inoculated into SDC liquid medium and cultured overnight. Subsequently, the culture medium was transferred to fresh SDC medium and incubated at 25°C until reaching the early logarithmic phase (OD_{600} ~0.1). SAH was then added to the medium to a final concentration of 1 mM, and cells were harvested at both the mid-logarithmic phase (OD_{600} ~1.0) and early stationary phase (OD_{600} ~4.0). WT and *tor1*Δ cells were cultured in the same manner and used as controls. Likewise, *sah1-1* and F13 mutants were grown to the mid-logarithmic phase (OD_{600} ~1.0) and early stationary phase (OD_{600} ~4.0) and collected for further analysis. For experiments involving DZNep, the compound was introduced at the specified final concentration upon transfer to fresh SDC medium and cells were harvested at both the mid-logarithmic phase (OD_{600} ~1.0) and early stationary phase (OD_{600} ~4.0). In contrast, for the X2180 strain, cells were grown to either the mid-logarithmic phase (OD_{600} ~1.0) or the early stationary phase (OD_{600} ~7.0) and then collected for further analysis.

All measurements were performed using a FACS Calibur (Becton-Dickinson). MMP analysis was conducted using 3,3′-Dihexyloxacarbocyanine Iodide ($DiOC_6(3)$) (Molecular Probes, D273), as previously described (10). For MMP measurement, cells were collected by centrifugation (1 × 10^7 cells), resuspended in 1 ml of fresh SDC medium containing 200 nM $DiOC_6(3)$, and incubated at 25°C for 30 minutes with shaking. Following incubation, the cells were washed twice with 1 ml PBS, and the fluorescence intensity was measured in the FL1 channel.

Superoxide analysis in yeast cells using dihydroethidium (DHE) (Molecular Probes, D1168) was performed as previously described (11). The cells were harvested by centrifugation at 1 × 10^7 cells, the precipitates were resuspended in 500 μL PBS containing 50 μM DHE, and incubated at 25°C for 10 minutes with shaking. Subsequently, the cells were washed twice with 1 ml of PBS, and the fluorescence intensity was measured using the FL2 channel.

All data were represented as the average of three biological replicates. At least three technical replicates were performed, and similar outcomes were obtained.

To ensure reproducible measurements, the MMP and ROS levels were primarily analyzed during the mid-logarithmic phase (OD_{600} ~1.0). Similar trends were observed across the early-to-mid-logarithmic phase (OD_{600} ~0.2), which was the growth stage utilized in the majority of the subsequent experiments.

Measurement of oxygen consumption rate

Cells were inoculated into SDC liquid medium and cultured overnight. Subsequently, the cultures were transferred to fresh SDC medium and incubated at 25°C until reaching an early logarithmic phase (OD_{600} ~0.1). SAH was then added to the SDC liquid medium to a final concentration of 1 mM, and cells were collected at the early-to-mid-logarithmic phase (OD_{600} ~0.2). WT and *tor1*Δ cells cultured to the same growth phase (OD_{600} ~0.2) served as controls. Following harvest, the cells were resuspended in fresh SDC liquid medium at a density of 1×10^7 cells/ml. The cells were treated with 1 µM Antimycin A (LKT-Laboratories, Inc., A5378), an inhibitor of respiratory chain complex III, as a negative control for impaired respiration, or with 5 µM FCCP (carbonyl cyanide 4-(trifluoromethoxy) phenylhydrazone) (Selleck, S8276) as a positive control. Subsequently, MitoXpress Xtra reagent (Agilent, MX-400-04) was added to 90 µL of the cell suspension mixed with 10 µL of the reagent, transferred into a well of a flat-bottom black clear-bottom 96-well plate (NEST, WNB701401), and overlaid with mineral oil. Time-resolved fluorescence signals were recorded every 1 minute for 90 minutes at 25°C using an Infinite 200 Pro plate reader (Tecan) with excitation and emission wavelengths of 380 and 650 nm, respectively. Signals were acquired with a delay time of 30 µs and gate time of 100 µs, and oxygen consumption rates were determined from the initial slopes of the resulting fluorescence profiles (µs/min). Relative oxygen consumption rates were calculated by assigning a value of 1 to untreated WT cells.

Extraction of the intracellular metabolite from yeast

Metabolite extraction was conducted using a previously described method (12). Cells were cultured to the early-to-mid-logarithmic phase (OD_{600} ~0.2) in SDC liquid medium at 25°C. Subsequently, the cells were harvested from the culture medium (OD_{600} = 30) and filtered through a 0.45 µm pore size filter. The internal cationic and anionic standards were methionine sulfone and 2-morpholinoethanesulfonic acid, respectively. The lyophilized samples were dissolved in 50 µL Milli-Q-grade water and subjected to capillary electrophoresis-time-of-flight mass spectrometry (CE-TOFMS).

Method for metabolome analysis

Instrumentation: All capillary electrophoresis mass spectrometry (CE-MS) experiments were conducted using Agilent 7100 CE capillary electrophoresis (Agilent Technologies, Waldbronn, Germany), Agilent 6230 LC/MSD TOF system (Agilent Technologies, Palo Alto, CA, USA), an Agilent1100 series binary HPLC pump, and the G1603A Agilent CE-MS adapter- and G1607A Agilent CE-ESI-MS sprayer kit. For anionic metabolite analysis, the original Agilent stainless Electrospray ionization (ESI) needle was replaced with the Agilent G7100-60041 platinum ESI needle (13). Agilent MassHunter Workstation was used to conduct system control and data acquisition, and data analysis was done using the Keio MasterHands software.

Cationic metabolite analysis by CE-MS: Separations were conducted in a fused silica capillary (50 µm i.d. × 100 cm total length) filled with 1 M formic acid as the electrolyte (12, 14). About 5 nl sample solution was injected at 50 mbar for five seconds, and 30 kV of voltage was applied. The capillary temperature was maintained at 20°C and the sample tray was cooled below 5°C. Methanol-water (50% v/v) containing 0.01 µM Hexakis (2,2-difluoroethoxy) phosphazene was delivered as the sheath liquid at 10 µl/min. ESI-time-of-flight mass spectrometry (ESI-TOFMS) was performed in the positive ion mode and the capillary voltage was set at 4,000 V. A flow rate of heated dry nitrogen gas (heater temperature 300°C) was maintained at 7 psig. In TOFMS, the fragmentor-, skimmer-, and Oct RFV voltage were set at 75 V, 50 V, and 500 V, respectively. Automatic recalibration of each acquired spectrum was conducted using reference masses of reference standards. The ^{13}C isotopic ion of a protonated methanol dimer ($[2MeOH + H]^+$, m/z 66.0631) and Hexakis(2,2-difluoroethoxy)phosphazene ($[M + H]^+$, m/z 622.0290) provided the lock mass for exact mass measurements (15).

Anionic metabolite analysis by CE-MS: A commercially available COSMO(+) (chemically coated with cationic polymer) capillary (50 µm i.d. × 105 cm total length) (Nacalai Tesque, Kyoto, Japan) was used with a 50 mM ammonium acetate solution (pH 8.5) as the electrolyte (13, 15). Sample solution (30 nl) was injected at 50 mbar for 30 sec and -30 kV of voltage was applied. Ammonium acetate (5 mM) in 50% methanol-water (v/v) containing 0.01 µM Hexakis(2,2-difluoroethoxy)phosphazene was delivered as the sheath liquid at 10 µl/min. ESI-TOFMS was conducted in the negative ion mode; the capillary voltage was set at 3,500 V. For TOFMS, the fragmentor-, skimmer-, and Oct RFV voltage were set at 100 V, 50 V, and

500 V, respectively. Automatic recalibration of each acquired spectrum was conducted using reference masses of reference standards, i.e., ^{13}C isotopic ion of deprotonated acetic acid dimer ($[2CH_3COOH-H]^-$, m/z 120.0384), and Hexakis + deprotonated acetic acid (m/z 680.03554) provided the lock mass for exact mass measurements

Statistical analysis: MetaboAnalyst software ver. 5.0 was used for statistical analysis (16). In instances where metabolites were not detected (N.D.) in only one condition, the missing values were imputed as zero. Metabolites were excluded from the analysis if missing values (N.D.) were present in both conditions being compared. Data scaling was conducted by mean-centered and divided by the standard deviation of each variable. The processed metabolomics data used to generate Figs. 2A–E and S3–S8 are provided in Dataset S2. The complete normalized and unfiltered metabolomics dataset is provided in Dataset S3.

Determination of SAM and SAH

SAM and SAH were extracted as previously described (17). Cells were cultured in SGC liquid medium at 25°C until they reached the early-to-mid-logarithmic phase (OD_{600} ~0.2). The cells were harvested (total OD600 of 15), washed twice with 20 ml of cold water, and then extracted with 1 ml of 10% perchloric acid (Fujifilm Wako Pure Chemical Corporation,160-05755) for 1 hour at room temperature. The supernatant was diluted with Milli-Q-grade water (Merck, Direct-Q UV8), and the samples were filtered through a 0.2-µm membrane filter (ADVANTEC, 13CP020AN) for capillary electrophoresis (CE). SAM and SAH were analyzed by capillary electrophoresis using an Agilent Capillary Ion Analyzer G1602A (Agilent) with an Agilent HPCE standard capillary (72-cm total length and 75-µm i.d.). SAM and SAH contents were expressed as nanomoles per milligram of dry cell weight.

Growth Conditions for Metabolome Analysis Using [methyl-^{13}C]Met

WT cells and the *GAL1-YBT1* strain were preincubated in SR+[methyl-^{13}C]Met liquid medium containing 20 mg/L [methyl-^{13}C]Met (Cambridge Isotope Laboratories, CLM-206-1) as the sole Met source at 25°C overnight. The SR medium had the same composition as the SDC medium but contained 2% (w/v) raffinose (Nacalai Tesque, 30001-15) instead of glucose. The cells were then grown in fresh SR+[methyl-^{13}C]Met liquid medium until the cell density reached the early logarithmic phase (OD_{600} ~0.1). These procedures were designed to ensure that the only external source of Met was [methyl-^{13}C]Met. The *GAL1-YBT1* strain culture was divided into two parts: one was treated with sterilized water, while the other was supplemented with SAM to achieve a final concentration of 1 mM. Both aliquots were incubated for 1 hour. Subsequently, galactose was added to each culture to reach a final concentration of 2%, followed by an additional 6-hour incubation. The WT strain served as a control; sterilized water was added, followed by a 1-hour incubation, 2% galactose was introduced, and incubation continued for another 6 hours. The [methyl-^{13}C]Met and [methyl-^{13}C]SAM contents were expressed as nmol per mg of dry cell weight (DCW).

Extraction of intracellular [methyl-^{13}C]Met and [methyl-^{13}C]SAM from yeast

Intracellular [methyl-^{13}C]Met and [methyl-^{13}C]SAM were extracted as previously described (18). The cells were harvested (total OD_{600} = 15), washed twice with 20 ml of cold water, and then extracted with 1 ml of 10% perchloric acid (Fujifilm Wako Pure Chemical Corporation, 160-05755) for one hour at room temperature. The supernatant was diluted with Milli-Q-grade water, and the samples were filtered through a 0.2-µm membrane filter (ADVANTEC, 13CP020AN) for CE-TOFMS.

GFP-Atg8 processing assay

Cells containing the GFP-Atg8 expressing plasmid were cultivated to the early-to-mid-logarithmic phase (OD_{600} ~0.2) in liquid SD or SG medium lacking uracil (SD-Ura or SG-Ura) at 25ºC. For experiments involving DZNep supplementation, cells were grown to the mid-logarithmic phase (OD_{600} ~1.0) in liquid SD-Ura medium containing DZNep at a final concentration of 100 or 500 µM at 25ºC. Samples were collected at 0 minute prior to the induction of nitrogen starvation. Subsequently, the cells were washed twice with liquid SD or SG medium lacking ammonium sulfate and amino acids, except for adenine, histidine, tryptophan, and leucine (SD-N or SG-N). Autophagy was induced by transferring the cells to fresh liquid SD-N or SG-N. In experiments with 100 µM DZNep, the compound was added to maintain a final concentration of 100 µM after shifting to the SD-N medium.

Nitrogen starvation was induced over a period of 0 to 240 minutes at 25ºC. Cell growth was halted by the addition of trichloroacetic acid (TCA; Sigma-Aldrich, 30-3890-5) to a final concentration of

approximately 9%, followed by incubation on ice for a minimum of 5 minutes before pelleting. The cells were collected by centrifugation, washed twice with cold acetone (Nacalai Tesque, 00310-95), and dried using a centrifugal evaporator (WakenBtech Co., Ltd., PV-1200). The pellets were resuspended in 100 µL of urea buffer [50 mM Tris (Sigma-Aldrich, T1378) [pH 7.5], 1 mM EDTA (Sigma-Aldrich, 09-1420-5), 6 M urea (Kanto Chemical, 43009-01), 1% SDS (Nacalai Tesque, 08933-05), 1 mM PMSF (Sigma-Aldrich, P7626), 1× complete protease inhibitor cocktail (Roche, 11873580001), and 0.1% Tween 20 (Bio-Rad, 1706531)] and lysed by vortexing with an equal volume of glass beads at room temperature, followed by heating for 10 minutes at 65ºC. Subsequent analysis was performed using SDS-PAGE and immunoblotting with anti-GFP antibody (Roche, 11814460001, 1:1000) and anti-PSTAIR antibody (Novus Bio., NB120-10345, 1:1000). ImageJ was used to quantify the signals for western blotting results, and GraphPad Prism 9 was used for statistical analysis.

GFP signals were normalized with respect to Cdc28 signals, and relative intensities were determined by assigning a value of 100 to the 240-minute signal intensity of the control condition (either WT or untreated cells).

Detection of Rps6 activation

Cells were inoculated into SDC or SGC liquid media and cultured overnight. Subsequently, the cultures were transferred to fresh SDC or SGC media. When supplemented with DZNep, DZNep was added at the time of transfer to the SDC liquid medium to achieve final concentrations of 100 or 500 µM. The cells were then incubated at 25°C until the cell density reached the early-to-mid-logarithmic phase (OD_{600} ~0.2).

Subsequently, the cells corresponding to 2×10^7 cells were harvested and washed with 1 ml of TEG +PPi buffer [50 mM Tris (Sigma-Aldrich, T1378)-HCl (Nacalai Tesque, 18321-05), 1 mM EDTA (Sigma-Aldrich, 09-1420-5) [pH 7.5], 10% glycerol (Nacalai Tesque, 17018-25), 30 mM NaCl (Nacalai Tesque, 31320-76), 10 mM NaF (Fujifilm Wako Pure Chemical Corporation, 192-01972), 1 mM sodium orthovanadate (Na3VO4) (Fujifilm Wako Pure Chemical Corporation, 198-09752), 1 mM PMSF (Sigma-Aldrich, P7626), 1 mM DTT (Nacalai Tesque, 14112-81), 1× complete protease inhibitor cocktail (Roche, 11873580001), and 0.1% Tween 20 (Bio-Rad, 1706531)]. The precipitate was then frozen in liquid nitrogen and stored at -80°C. The frozen precipitate was dissolved on ice, gently resuspended in SDS-PAGE sample buffer, and boiled for 10 minutes. The eluted proteins were resolved by SDS-PAGE and detected using anti-Phospho-S6 Ribosomal Protein (Ser235/236) antibody (Cell Signaling Tech., 4858, 1:1000) and anti-PSTAIR antibody (Novus Bio., NB120-10345, 1:1000). ImageJ was employed to quantify signals for Western blotting results, and GraphPad Prism 9 was utilized for statistical analysis.

Phospho-Rps6 signals were normalized to Cdc28 signals, and relative intensities were calculated by assigning a value of 1 to the control (WT or untreated) cells.

Detection of Snf1/AMPK Activation

Cells were inoculated into SDC or SGC liquid medium and cultured overnight. Subsequently, the cultures were transferred to fresh SDC or SGC medium. When supplemented with DZNep, DZNep was added at the time of transfer to the SDC liquid medium to achieve a final concentration of 100 or 500 µM. The cells were then incubated at 25°C until the cell density reached the early-to-mid-logarithmic phase (OD_{600} ~0.2). Cultures corresponding to 1.5×10^7 cells were boiled for 5 minutes, cooled to room temperature, and the cells were harvested by centrifugation. The resulting pellets were resuspended in 150 µL of 1× TE buffer [10 mM Tris-HCl, 1 mM EDTA (pH 7.5)] and 150 µL of 0.2 M NaOH. After 5 minutes of incubation at room temperature, the pellets were collected and gently resuspended in SDS-PAGE sample buffer, followed by boiling for 5 minutes. The eluted proteins were resolved using SDS-PAGE and detected with a Phospho-AMPKα (Thr172) antibody (Cell Signaling Tech., 2535, 1:1000). To detect the total Snf1 protein, a polyhistidine antibody (Sigma-Aldrich, H1029) was used. ImageJ software was used to quantify the signals from the western blotting results, and GraphPad Prism 9 was used for statistical analysis.

Phospho-Snf1 signals were normalized to Snf1 signals, and relative intensities were calculated by assigning a value of 1 to the control (WT or untreated) cells.

Quantitative reverse-transcription PCR (qPCR)

Cells were inoculated into SDC liquid medium and cultured overnight. Subsequently, the cultures were transferred to a fresh SDC medium. When supplemented with DZNep, DZNep was added at the time of transfer to the SDC liquid medium to achieve a final concentration of 100 µM. The cells were then incubated at 25°C until the early logarithmic phase (OD_{600} ~0.1). At this time, SAH was added to the SDC

liquid medium to achieve a final concentration of 0.5 mM, and cells were harvested upon reaching the early-to-mid-logarithmic phase (OD_{600} ~0.2). WT cells were cultured to the early-to-mid-logarithmic phase (OD_{600} ~0.2) as controls. In the DZNep-treated samples, cells were harvested at the mid-logarithmic phase (OD_{600} ~1.0), and WT cells harvested at the mid-logarithmic phase (OD_{600} ~1.0) were used as controls. Cells were collected by centrifugation (2×10^7 cells), and total RNA was isolated using the RNeasy Mini Kit (Qiagen, 74104) following the manufacturer's instructions. Genomic DNA in the samples was then removed using an RNase-Free DNase Set (Qiagen, 79254). qPCR was performed using the One Step TB Green® PrimeScript™ RT-PCR Kit (Perfect Real Time) (Takara, RR066A) on a LightCycler instrument (Roche Molecular Biochemicals). The reaction conditions were as follows: reverse transcription at 42°C for 5 minutes, followed by initial denaturation at 95°C for 10 s for 1 cycle; PCR amplification was then performed for 60 cycles at 95°C for 5 s and 60°C for 20 s. Transcript levels were normalized to those of *ACT1*, which was used as an internal reference gene. Relative expression levels were determined by assigning a value of 1 to the control (WT or untreated) cells. Data were analyzed using the LightCycler Software Version 3.5.28. The primer sequences are listed in Table S3.

SAM uptake assay

Isolation of the vacuolar membrane vesicles

The right-side-out vacuolar membrane vesicles of *S. cerevisiae* cells were isolated as described previously (2, 3, 19) with slight modifications. Vacuolar membrane vesicles for Figs. 4B and C were prepared from cells in the X2180-1B *ura3*Δ background; vesicles for Fig. 4B were prepared from both WT (STY3807) and *GPD-YBT1* (YMA245) strains, whereas those for Fig. 4C were prepared from the *GPD-YBT1* strain alone.

Briefly, yeast cells were cultured overnight in YPD medium to an OD_{600} of 1.5–2.0 (approximately 3×10^7 cells/mL) and harvested by centrifugation. Approximately 3×10^3 optical density (OD) of exponentially growing cells were incubated with gentle shaking for 10 minutes in the pretreatment buffer (0.1 M Tris-HCl, pH 7.5, 0.28% β-mercaptoethanol), then collected and resuspended in 1 M sorbitol containing 200 μg/mL Zymolyase-20T (Nacalai Tesque, 007663-91), and incubated at 30°C for 60 min with gentle shaking to convert them to spheroplasts. The spheroplasts collected by centrifugation were suspended in 6 volumes of Buffer A containing 10 mM MES-Tris (pH 6.9), 0.1 mM $MgCl_2$, 12% Ficoll ™ PM400 (Cytiva, 17030050), 1 mM PMSF, and EDTA-free protease inhibitor cocktail (Roche, 11836170001), and homogenized using a loosely fitting Dounce homogenizer to disrupt the cells. The 20 mL of disrupted cells were gently layered on top by 10 mL of Buffer A in ultracentrifuge tubes, and then centrifuged at 50,000 × *g* for 60 minutes at 4°C using a swing rotor. After centrifugation, the intact vacuoles were collected from the white layer floating on top. The collected vacuoles were resuspended in 20 mL of Buffer A and homogenized again with a loosely fitting homogenizer, and then layered on top with 10 mL of Buffer B (10 mM MES-Tris, pH 6.9, 0.5 mM $MgCl_2$, 8% Ficoll, 1 mM PMSF, EDTA-free protease inhibitor cocktail), and centrifuged as above. Intact vacuoles were collected and converted to vacuolar membrane vesicles by diluting and homogenizing them with Buffer C (10 mM MES-Tris, pH 6.9, 5 mM $MgCl_2$, 25 mM KCl) using a tightly fitting Dounce homogenizer. The vesicles were recovered as pellets by centrifugation at 37,000 × *g* for 30 minutes at 4°C.

ATP-dependent uptake of SAM into vacuolar membrane vesicles

To measure the uptake activity for SAM, vesicles (330 μg of protein) were mixed with or without 0.5 mM ATP in the assay mixture (891 μL) consisting of 25 mM MES-Tris (pH 6.9), 4 mM $MgCl_2$, and 25 mM KCl. After incubation of the mixture for 1 minute at 25˚C, the reaction was initiated by adding [^{14}C]SAM (Adenosyl-l-Methionine, S-[methyl-^{14}C]-) (final 0.1 mM; 1.48-2.22 GBq/mmol, Revvity, NEC363010UC) and then stopped by diluting a 300 μL sample with 5 ml of ice-cold wash buffer consisting of 25 mM MES-Tris (pH 6.9), 5 mM $MgCl_2$, and 25 mM KCl at indicated time points. Vesicles were recovered by vacuum filtration onto a 0.45-μm cellulose acetate filter membrane (Advantec, C045A293C). The filters were washed once with 5 ml of ice-cold wash buffer. The radioactivity remaining on the filters was determined using a xylene scintillator in a liquid scintillation counter. [methyl-^{14}C]SAM was purchased from Revvity (Yokohama, Japan).

For competitive inhibition assays, uptake measurements were performed in the presence of 0.1 mM [^{14}C]SAM with non-labeled SAH, SAM, or Met at final concentrations of 0.1, 0.2, 0.5, or 1 mM.

Structural modeling
The structure model was generated using SWISS-MODEL (https://swissmodel.expasy.org/) (20) with reference to known homologous protein structures (21).

Microscopy
To visualize vacuolar morphology, yeast cells were stained with the vital dye FM4-64 (Invitrogen, T13320). Cells were cultured to the logarithmic phase in SDC liquid medium at 25°C and then harvested by centrifugation. The collected cells were resuspended in YPD medium containing 40 µM FM4-64 at an OD600 of 20 and incubated at 25°C for 20 minutes. After staining, the cells were washed with 1 ml of PBS, transferred to fresh SDC medium, and incubated at 25°C for an additional 2 hours. Images were obtained using a Zeiss Axio Observer Z1 inverted microscope equipped with a Plan-Apochromat 63x/1.40 Oil DIC and an Axiocam 503 mono digital camera.

Statistical analysis and reproducibility
No statistical methods were used to determine the sample size. All experiments were not scored blindly. Metabolome experiments (Figs. 2A-E), and SAM uptake assay (Figs. 4B and C), and measurements of intracellular [methyl-^{13}C]Met and [methyl-^{13}C]SAM (Fig. 4K) were conducted in triplicate in independent experiments, and the data were analyzed using the means of the triplicate samples. All other experiments were performed in at least two technical replicates. *n* indicates the number of independent biological replicates. Appropriate statistical tests were used for each figure. Significance calculations were performed using GraphPad Prism software 9.0 (GraphPad Software, San Diego, CA, USA). Data are presented as mean ± S.E.M. or ± S.D.; *p*-values less than 0.05 were considered statistically significant.

Table S1. *Saccharomyces cerevisiae* strains used in this study.

Strain	Genotype	Source or reference
**W303-1A derivatives**		
BY5701	*MATa, ura3-1 leu2-3,112 trp1-1 ade2-1 his3-11,15 can1-100*	National BioResource Project/ Yeast Genetic Resource Center, Japan
YMA32	*tor1 Δ::kanMX4*	Ogawa *et al*. (22)
YMA501	*cyc3 Δ::kanMX6*	this study
YMM222	*sah1-1*	Mizunuma *et al*. (17)
F13	F13	Ogawa *et al*. (9)
YMA174	*sah1* D226Y T279I	this study
YMA202	*sah1* D226Y	this study
YMA145	*YBT1::GAL1pr-kanMX6*	this study
YMA265	*ybt1* K735M*::GAL1pr-kanMX6*	this study
YMA241	*ybt1 Δ::kanMX4*	this study
YMA280	*ybt1* K735M	this study
YMA176	*YBT1::GFP-kanMX6*	this study
YMA228	*ybt1* K735M*::GFP-kanMX6*	this study
YMA276	*YBT1::GAL1pr-kanMX6 tor1 Δ::kanMX4*	this study
YYK1	*atg7 Δ::kanMX4*	Ogawa *et al*. (22)
YMA268	*YBT1::GAL1pr-kanMX6 atg7 Δ::kanMX4*	this study
YTK22	*snf1 Δ::kanMX6*	Ogawa *et al*. (9)
YMA270	*YBT1::GAL1pr-kanMX6 snf1 Δ::kanMX6*	this study
YMA220	*YBT1::GPDpr-kanMX4*	this study
YMA226	*ybt1* K735M*::GPDpr-kanMX4*	this study
**X2180-1B derivatives**		
STY3807	*MAT α, SUC2 mal mel gal2 CUP1 ura3 Δ::loxP*	Sekito *et al*. (4)
YMA245	*ura3 Δ::loxP YBT1::GPDpr-kanMX4*	this study
**BY4742 derivatives**		
BY4742	*MAT α, his3 Δ1 leu2 Δ0 lys2 Δ0 ura3 Δ0*	Open Biosystems, Inc.
	avt1 Δ::kanMX4	Open Biosystems, Inc.
	avt2 Δ::kanMX4	Open Biosystems, Inc.
	avt3 Δ::kanMX4	Open Biosystems, Inc.
	avt4 Δ::kanMX4	Open Biosystems, Inc.
	avt5 Δ::kanMX4	Open Biosystems, Inc.
	avt6 Δ::kanMX4	Open Biosystems, Inc.
	avt7 Δ::kanMX4	Open Biosystems, Inc.
	vba1 Δ::kanMX4	Open Biosystems, Inc.
	vba2 Δ::kanMX4	Open Biosystems, Inc.
	vba3 Δ::kanMX4	Open Biosystems, Inc.
	vba4 Δ::kanMX4	Open Biosystems, Inc.
	ypq1 Δ::kanMX4	Open Biosystems, Inc.
	ypq2 Δ::kanMX4	Open Biosystems, Inc.
YMA307	*rtc2 Δ::kanMX4*	this study
	ers1 Δ::kanMX4	Open Biosystems, Inc.
	uga4 Δ::kanMX4	Open Biosystems, Inc.
	atg22 Δ::kanMX4	Open Biosystems, Inc.
	ycf1 Δ::kanMX4	Open Biosystems, Inc.
	bpt1 Δ::kanMX4	Open Biosystems, Inc.
	nft1 Δ::kanMX4	Open Biosystems, Inc.
	vmr1 Δ::kanMX4	Open Biosystems, Inc.
	ybt1 Δ::kanMX4	Open Biosystems, Inc.
	adp1 Δ::kanMX4	Open Biosystems, Inc.
	yol075c Δ::kanMX4	Open Biosystems, Inc.
	yhr032w Δ::kanMX4	Open Biosystems, Inc.
	ydr338c Δ::kanMX4	Open Biosystems, Inc.

Table S2. Growth media, chemical compositions, and corresponding figures used in this study.

Media	Composition	Application and corresponding figures
YPD	1% (w/v) yeast extract<br>2% (w/v) peptone<br>2% (w/v) glucose<br>400 mg/L adenine<br>200 mg/L uracil	**Dilution spot assay**: S2D<br>**CFU determination in CLS assays**: 1D, 1F, 1J, 1M, 3C–E, 3H, 4H–J, 4L, S1B, S2C, S2E, S11A–B<br>**Cell culture for vacuolar membrane vesicle preparation**: 4B–C
SDC	0.17% (w/v) yeast nitrogen base without amino acids and ammonium sulfate<br>0.5% (w/v) ammonium sulfate<br>2% (w/v) glucose<br>20 mg/L each of adenine, arginine, histidine, methionine, tryptophan, and uracil<br>30 mg/L each of isoleucine, leucine, lysine, and tyrosine<br>60 mg/L phenylalanine<br>150 mg/L valine	**Flow cytometry**: 1A–B, 1H–I, 1K–L, 3I–J, S1A<br>**Oxygen consumption assay**: 1C<br>**CLS assay**: 1D, 1F, 1J, 1M, 3D–E, S1B, S2C, S2E, S11A–B<br>**Metabolome analysis**: 2A–E, S3A–B, S4–S7, S8A–B<br>**Detection of Rps6 activation**: 2F, 2I<br>**Detection of Snf1/AMPK activation**: 2H, 2K<br>**Dilution spot assay**: 3A, 4D, S10C<br>**YBT1 expression analysis (RT-qPCR)**: 3F–G<br>**Ybt1-GFP localization**: 4A
SD-Ura	Same as SDC, except that uracil was omitted	**GFP-Atg8 processing assay (preculture)**: 2G, 2J, S9
SD-N	0.17% (w/v) yeast nitrogen base without amino acids and ammonium sulfate<br>2% (w/v) glucose<br>20 mg/L each of adenine, histidine, and tryptophan<br>30 mg/L leucine<br>Supplements were added to satisfy the auxotrophic requirements of W303-derived strains.	**GFP-Atg8 processing assay (nitrogen starvation)**: 2G, 2J, S9
SD-Met	Same as SDC, except that methionine was omitted	**Dilution spot assay**: 3A, S10B
SGC	Same as SDC, except that 2% (w/v) galactose was used instead of glucose	**Dilution spot assay:** 3A, 4D, S10C<br>**CLS assay**: 3C, 3H, 4H–J, 4L<br>**Determination of SAM and SAH**: 3B<br>**Detection of Rps6 activation**: 4E<br>**Detection of Snf1/AMPK activation**: 4G
SG-Met	Same as SGC, except that methionine was omitted	**Dilution spot assay**: 3A
SG-Ura	Same as SGC, except that uracil was omitted	**GFP-Atg8 processing assay (preculture)**: 4F
SG-N	Same as SD-N, except that 2% (w/v) galactose was used instead of glucose	**GFP-Atg8 processing assay (nitrogen starvation)**: 4F
SR	Same as SDC, except that 2% (w/v) raffinose was used instead of glucose	**Stable-isotope tracing with [methyl-^{13}C]Met**: 4K

Table S3. Oligonucleotides used in this study

**Oligonucleotides**	**Sequence**
TOR1 deletion Forward primer	5'-TTTGTATTTGAAGGTGCAGCTGTGG-3′
TOR1 deletion Reverse primer	5'-CTACGAACACGTTTTGGTGATGAAG-3′
YBT1 deletion Forward primer	5'-TGTGCGCATCTGCAAAGAACGTACG-3′
YBT1 deletion Reverse primer	5'-GCAGTTATCCACCAACTCGGGTAAC-3′
CYC3 deletion Forward primer	5'-TAGCATACACATACGAGTAGCACGAATAAAGCAGAGCAAATACAACAGAAACTACAAAAACGGATCCCCGGGTTAATTAA-3'
CYC3 deletion Reverse primer	5'-GAATTCGAGCTCGTTTAAACCTAGGACATCATTATTTCTTACTTGGCTATTTACACGTACTTACGCTGGCTGTATATCAT-3'
RTC2 deletion Forward primer	5'-GTTATAACCCAGACGTAATAGCCTCAAGCGAGCATCCCTAAATTTCTGCCCGGATCCCCGGGTTAATTAA-3′
RTC2 deletion Reverse primer	5'-ATATGAGTAAGTTTGAGTTTTAAGTGGAAAGATTAATAGAAGCCAACTAGGAATTCGAGCTCGTTTAAAC-3′
kanMX6-GAL1 promoter-YBT1 Forward primer	5'-CTTTCCTAGATTTTGTACTTTTTAGAAGAGCTCATCAAGGGGGGAAAAGCTACGGAGAACGAATTCGAGCTCGTTTAAAC-3′
kanMX6-GAL1 promoter-YBT1 Reverse primer	5'-AGTTACGTCATCGTAAAACCAAAACCGATGGTCAGGTCTCGTTGAATTGAGTACGTGATGCATTTTGAGATCCGGGTTTT-3′
kanMX4-GPD promoter-YBT1 Forward primer	5'-CTTTCCTAGATTTTGTACTTTTTAGAAGAGCTCATCAAGGGGGGAAAAGCTACGGAGAACCGTACGCTGCAGGTCGAC-3′
kanMX4-GPD promoter-YBT1 Reverse primer	5'-TTACGTCATCGTAAAACCAAAACCGATGGTCAGGTCTCGTTGAATTGAGTACGTGATGCATCGATGAATTCTCTGTCG-3′
YBT1-GFP-kanMX6 Forward primer	5'-GATATCCTAATTGAATTGGCTAAAAAAGCCTTTGTGGAAAAATTGAACTCTAAAAAGGACCGGATCCCCGGGTTAATTAA-3′
YBT1-GFP-kanMX6 Reverse primer	5'-ATACATATATATATATATATATATATATATATATATATATATACTTTAGCATCGAAACAGGAATTCGAGCTCGTTTAAAC-3′
SAH1 sgRNA for SpCas9 Forward primer	5'-GGAGGTAACCCTGATGAGTCCGTGAGGACGAAACGAGTAAGCTCGTCGGTTACGGTGATGTCGGTAA-3′
SAH1 sgRNA for SpCas9 Reverse primer	5'-AAACTTACCGACATCACCGTAACCGACGAGCTTACTCGTTTCGTCCTCACGGACTCATCAGGGTTAC-3′
Donor fragment SAH1 D226Y Forward primer	5'-TAAGAGAGCCACTGATGTCATGTTGGCTGGTAAGGTTGCCGTTGTTGCTGGTTACGGTTATGTCGGTAAG-3′
Donor fragment SAH1 D226Y Reverse primer	5'-TTCGGTAACCAAGACACGAGCACCCATTCCTCTTAAGGCAGCAGCACAACCCTTACCGACATAACCGTAA-3′
YBT1 sgRNA for SpCas9 Forward primer	5'-GGAGTTACCACTGATGAGTCCGTGAGGACGAAACGAGTAAGCTCGTCTGGTAAGACATCCCTACTAA-3′
YBT1 sgRNA for SpCas9 Reverse primer	5'-AAACTTAGTAGGGATGTCTTACCAGACGAGCTTACTCGTTTCGTCCTCACGGACTCATCAGTGGTAA-3′
Donor fragment YBT1 K735M Forward primer	5'-TCAAAACTGGTAAACTAAATGTCGTTATTGGCCCCACTGGTTCTGGTATGACATCCCTATTGATGGCATT-3′
Donor fragment YBT1 K735M Reverse primer	5'-CTAGAGCTGGGACGACAACCTTACCATTGAGTAGATACATTTCACCTAATAATGCCATCAATAGGGATGT-3′
ACT1 qPCR-F	5'-TTGGATTCCGGTGATGGTGTTACT-3'
ACT1 qPCR-R	5'-TGAAGAAGATTGAGCAGCGGTTTG-3'
YBT1 qPCR-F	5'-CGATGAGGAGTCCACTTCTTCTGCC-3'
YBT1 qPCR-R	5'-GGTATAACAGCGCCAGTGCGACAAC-3'

SI References

1. M. S. Longtine *et al.*, Additional modules for versatile and economical PCR-based gene deletion and modification in Saccharomyces cerevisiae. *Yeast* **14**, 953–961 (1998).
2. M. Ishimoto, N. Sugimoto, T. Sekito, M. Kawano-Kawada, Y. Kakinuma, ATP-dependent export of neutral amino acids by vacuolar membrane vesicles of Saccharomyces cerevisiae. *Biosci. Biotechnol. Biochem.* **76**, 1802–1804 (2012).
3. M. Kawano-Kawada *et al.*, A PQ-loop protein Ypq2 is involved in the exchange of arginine and histidine across the vacuolar membrane of Saccharomyces cerevisiae. *Sci. Rep.* **9**, 15018 (2019).
4. T. Sekito *et al.*, Vacuolar transporter Avt4 is involved in excretion of basic amino acids from the vacuoles of Saccharomyces cerevisiae. *Biosci. Biotechnol. Biochem.* **78**, 969–975 (2014).
5. S. Okada, G. Doi, S. Nakagawa, E. Kusumoto, T. Ito, Simple-to-use CRISPR-SpCas9/SaCas9/AsCas12a vector series for genome editing in Saccharomyces cerevisiae. *G3 (Bethesda)* **11** (2021).
6. Y. Naito, K. Hino, H. Bono, K. Ui-Tei, CRISPRdirect: software for designing CRISPR/Cas guide RNA with reduced off-target sites. *Bioinformatics* **31**, 1120–1123 (2015).
7. P. Fabrizio, V. D. Longo, The chronological life span of Saccharomyces cerevisiae. *Aging Cell* **2**, 73–81 (2003).
8. P. Fabrizio, V. D. Longo, The chronological life span of Saccharomyces cerevisiae. *Methods Mol. Biol.* **371**, 89–95 (2007).
9. T. Ogawa *et al.*, Stimulating S-adenosyl-l-methionine synthesis extends lifespan via activation of AMPK. *Proc. Natl. Acad. Sci. U. S. A.* **113**, 11913–11918 (2016).
10. Y. Pan, G. S. Shadel, Extension of chronological life span by reduced TOR signaling requires down-regulation of Sch9p and involves increased mitochondrial OXPHOS complex density. *Aging (Albany N. Y.)* **1**, 131–145 (2009).
11. N. D. Bonawitz, M. S. Rodeheffer, G. S. Shadel, Defective mitochondrial gene expression results in reactive oxygen species-mediated inhibition of respiration and reduction of yeast life span. *Mol. Cell. Biol.* **26**, 4818–4829 (2006).
12. T. Soga *et al.*, Quantitative metabolome analysis using capillary electrophoresis mass spectrometry. *J. Proteome Res.* **2**, 488–494 (2003).
13. T. Soga *et al.*, Metabolomic profiling of anionic metabolites by capillary electrophoresis mass spectrometry. *Anal. Chem.* **81**, 6165–6174 (2009).
14. T. Soga *et al.*, Differential metabolomics reveals ophthalmic acid as an oxidative stress biomarker indicating hepatic glutathione consumption. *J. Biol. Chem.* **281**, 16768–16776 (2006).
15. K. Satoh *et al.*, Global metabolic reprogramming of colorectal cancer occurs at adenoma stage and is induced by MYC. *Proc. Natl. Acad. Sci. U. S. A.* **114**, E7697–e7706 (2017).
16. Z. Pang *et al.*, MetaboAnalyst 5.0: narrowing the gap between raw spectra and functional insights. *Nucleic Acids Res.* **49**, W388–w396 (2021).
17. M. Mizunuma, K. Miyamura, D. Hirata, H. Yokoyama, T. Miyakawa, Involvement of S-adenosylmethionine in G1 cell-cycle regulation in Saccharomyces cerevisiae. *Proc. Natl. Acad. Sci. U. S. A.* **101**, 6086–6091 (2004).
18. S. A. Christopher, S. Melnyk, S. J. James, W. D. Kruger, S-adenosylhomocysteine, but not homocysteine, is toxic to yeast lacking cystathionine beta-synthase. *Mol. Genet. Metab.* **75**, 335–343 (2002).
19. Y. Ohsumi, Y. Anraku, Active transport of basic amino acids driven by a proton motive force in vacuolar membrane vesicles of Saccharomyces cerevisiae. *J. Biol. Chem.* **256**, 2079–2082 (1981).
20. A. Waterhouse *et al.*, SWISS-MODEL: homology modelling of protein structures and complexes. *Nucleic Acids Res.* **46**, W296–w303 (2018).
21. Y. Kusakabe *et al.*, Structural insights into the reaction mechanism of S-adenosyl-L-homocysteine hydrolase. *Sci. Rep.* **5**, 16641 (2015).
22. T. Ogawa *et al*., S-adenosyl-L-homocysteine extends lifespan through methionine restriction effects. *Aging Cell* **21**, e13604 (2022).

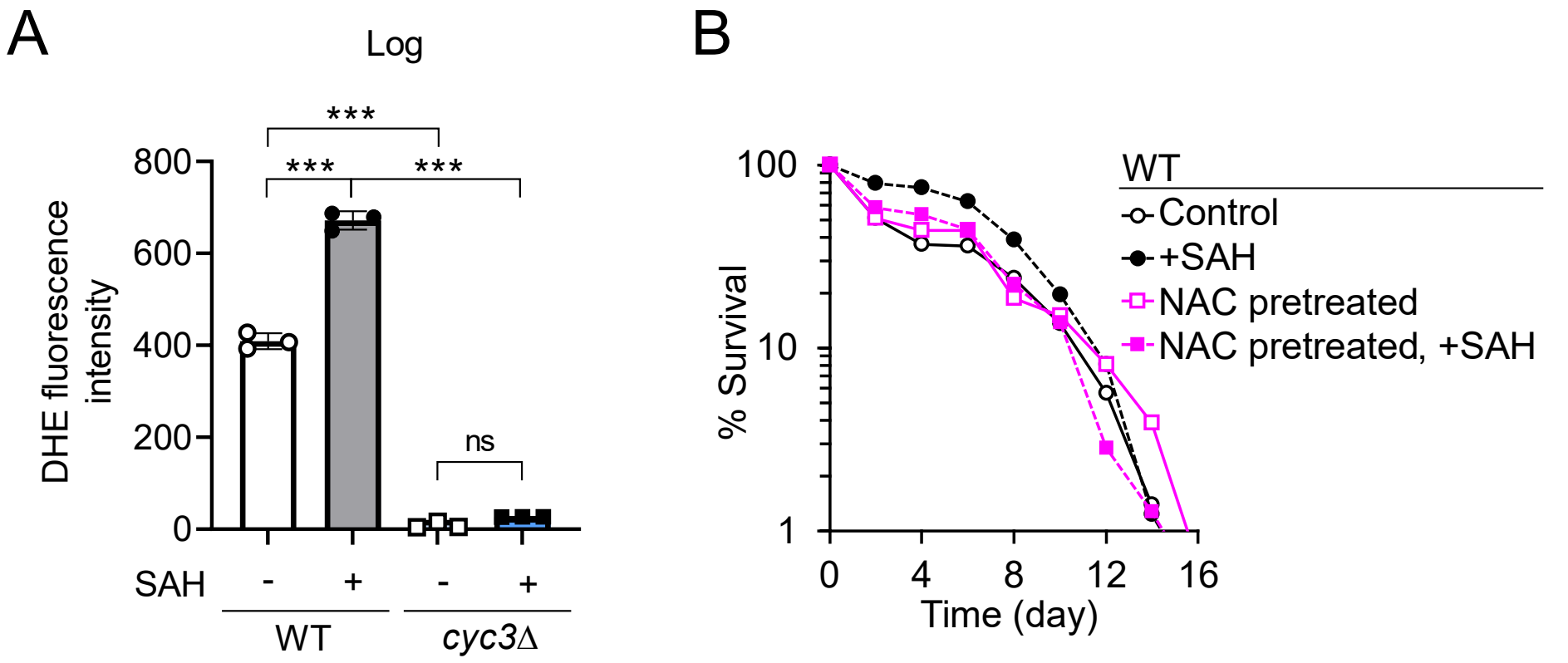

Fig. S1. SAH generates mitochondria-derived ROS, related to Figs. 1B and D. (**A**) Flow cytometry analysis of ROS levels (*n* = 3). Wild-type (WT) (BY5701; W303-1A) and *cyc3*Δ (YMA501) cells were cultured in SDC medium at 25°C with or without 1 mM SAH. (**B**) CLS survival curves (*n* = 3). WT (BY5701) cells were cultured in SDC medium at 28°C with or without 1 mM SAH, following pretreatment with or without 10 mM NAC for 2 h and subsequent washout. Data are presented as mean ± SD. Statistical significance was determined using two-way ANOVA with Tukey's test (A) or two-way ANOVA for the survival curves (B). ****P* < 0.001; ns, not significant. Detailed statistics are provided in Dataset S1.

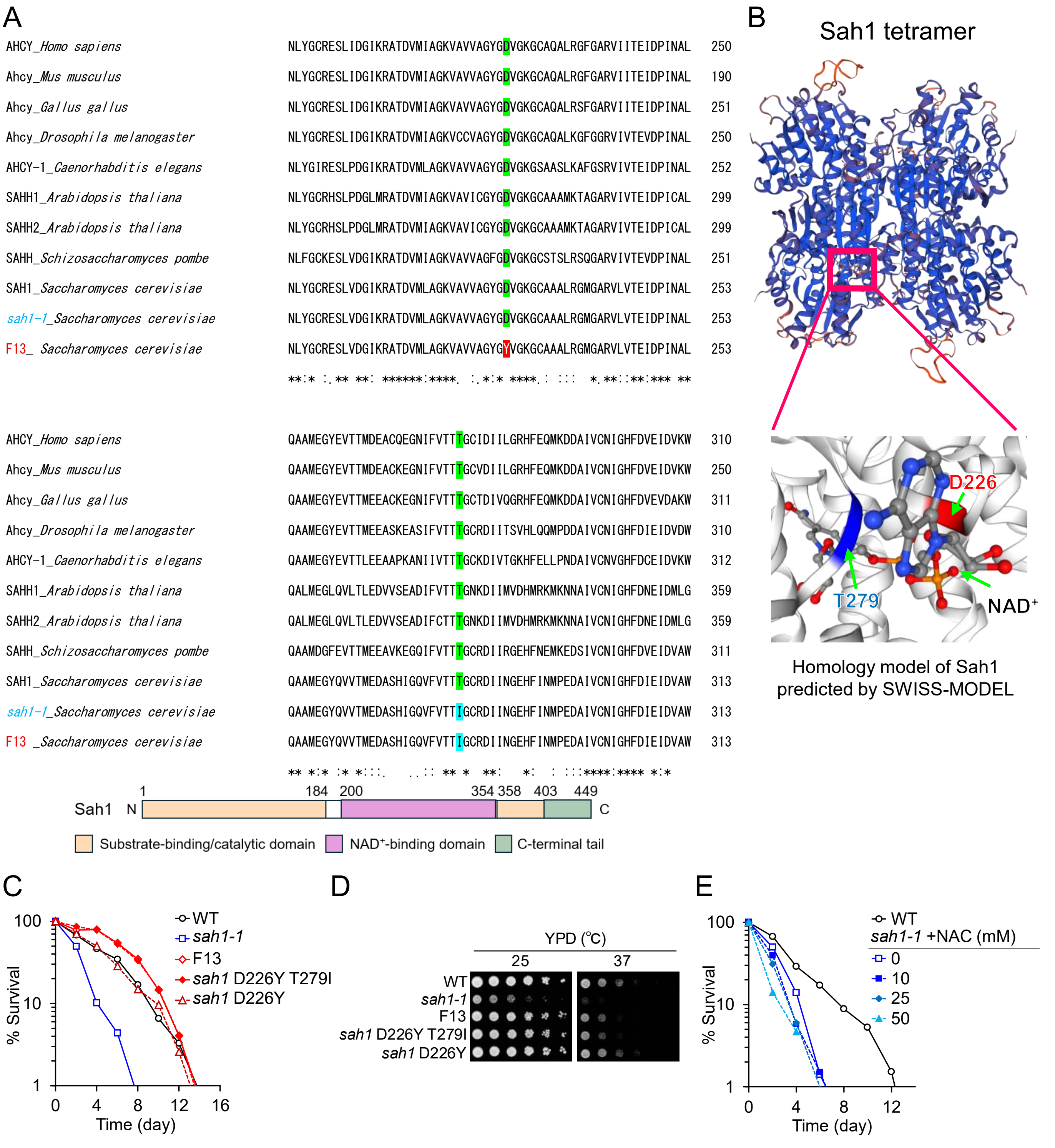

Fig. S2. The D226Y mutation in Sah1 mitigates the *sah1-1* phenotype, related to Figs. 1E–G. (**A**) Sequence alignment of the SAH hydrolase NAD$^+$-binding domain. Asterisks, colons, and periods denote identical, strongly conserved, and weakly conserved residues, respectively. (B) Homology model of the yeast Sah1 tetramer generated by SWISS-MODEL using the crystal structure of mouse SAHH (Protein Data Bank [PDB] ID: 5AXA) as a template, highlighting residues D226 (red) and T279 (blue) along with the NAD$^+$ molecule. (**C, E**) CLS survival curves (n = 3) in SDC medium at 28°C, using WT (BY5701; W303-1A), *sah1-1* (YMM222), F13 (F13), *sah1* D226Y T279I (YMA174), and *sah1* D226Y (YMA202) cells for (C), and WT (BY5701) and *sah1-1* (YMM222) cells with the indicated concentrations of NAC for (E). (**D**) Serial dilution growth assay of WT (BY5701), *sah1-1* (YMM222), F13 (F13), *sah1* D226Y T279I (YMA174), and *sah1* D226Y (YMA202) cells spotted on YPD plates at 25°C or 37°C. Growth at 25°C and 37°C was compared to more clearly distinguish the growth phenotypes of the *sah1* mutant strains. The significance of (C, E) was determined using a two-way ANOVA for the survival curves. Detailed statistics are provided in Dataset S1.

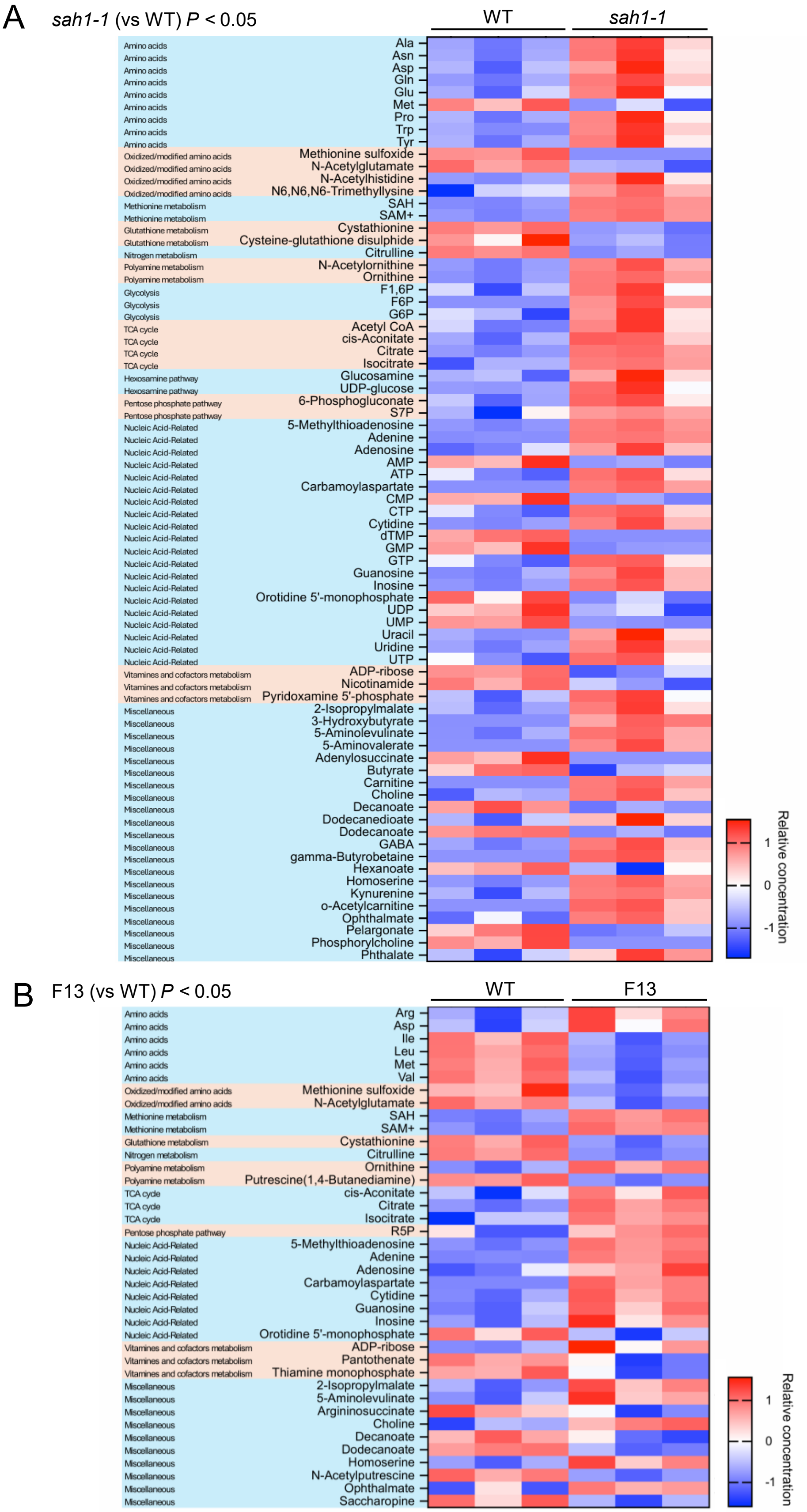

Fig. S3. Pathway categorization of differentially accumulated metabolites, related to Figs. 2A–E. Heatmap categorizing altered metabolites (P < 0.05, unpaired t-test) by metabolic pathways, based on the dataset from WT (BY5701; W303-1A), *sah1-1* (YMM222), and F13 (F13) cells cultured in SDC medium at 25°C. The detailed data are provided in Dataset S2.

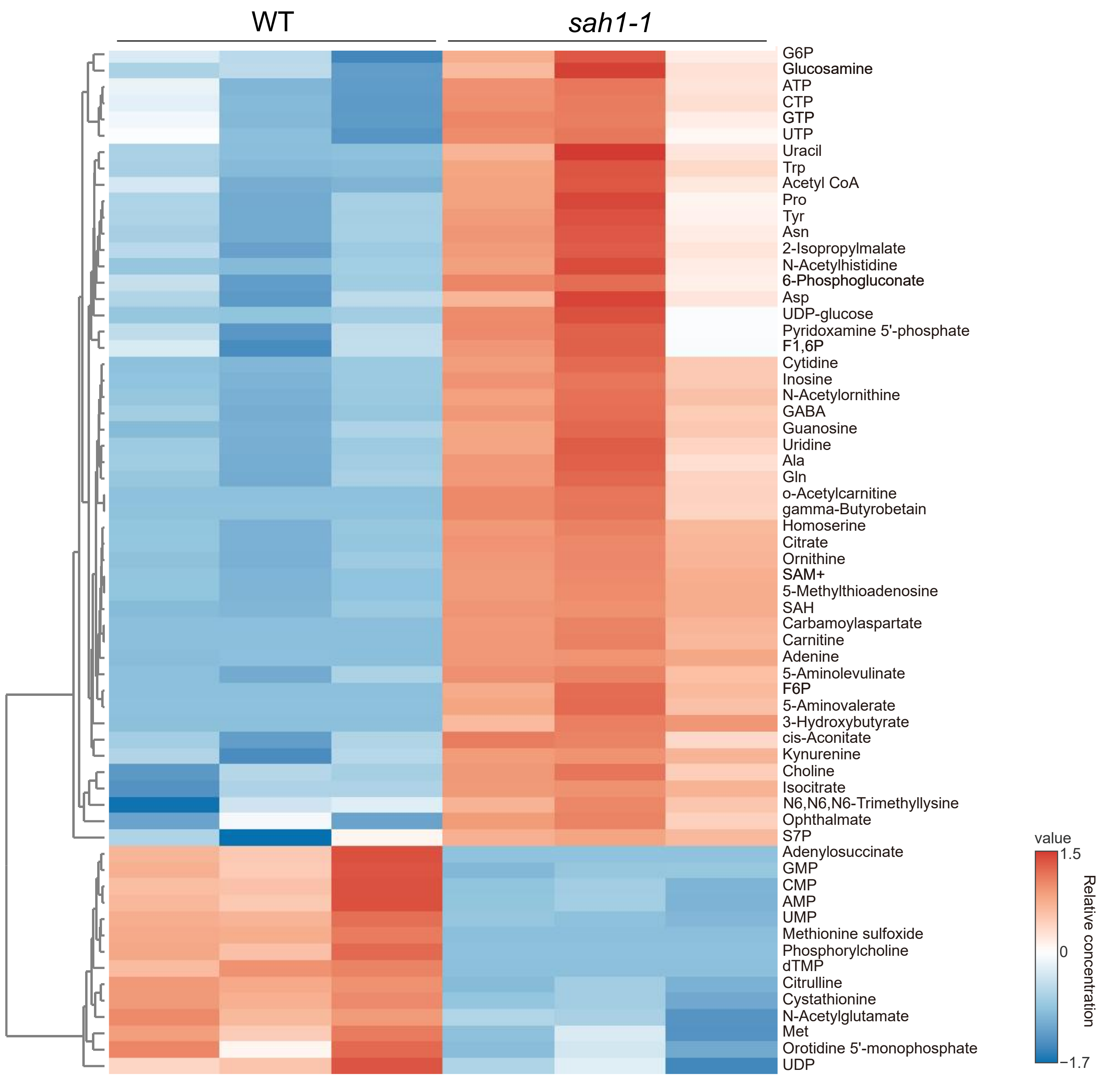

Fig. S4. Heatmap of differentially accumulated metabolites in *sah1-1* mutants, related to Fig. 2B. Hierarchical clustering of altered metabolites (P < 0.05, unpaired t-test; fold change ≥ 2 or ≤ 0.5) using Euclidean distance and average linkage based on WT (BY5701; W303-1A) and *sah1-1* (YMM222) cells cultured in SDC medium at 25°C. The detailed data are provided in Dataset S2.

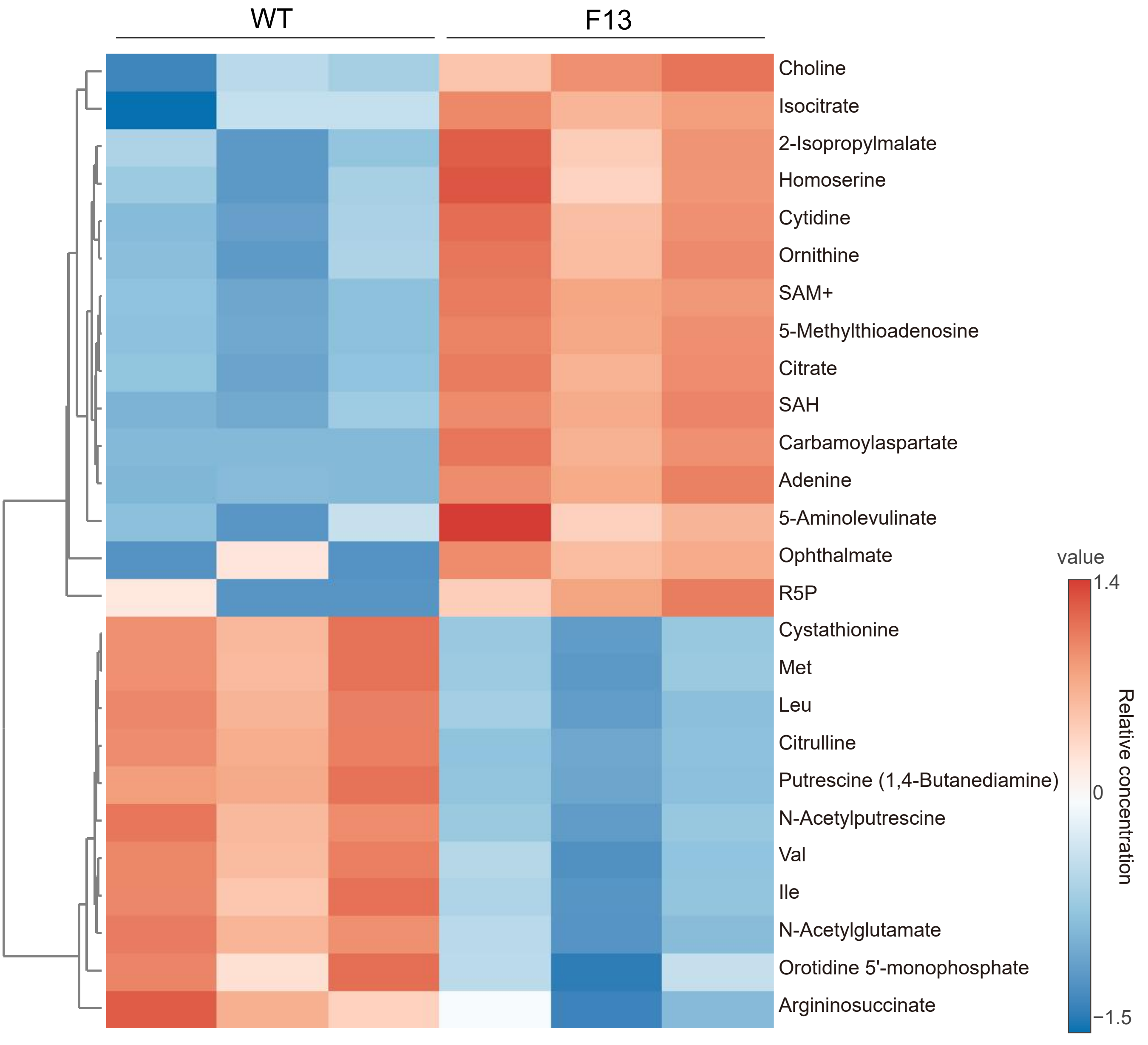

Fig. S5. Heatmap of differentially accumulated metabolites in F13 mutants, related to Fig. 2D. Hierarchical clustering of altered metabolites ($P < 0.05$, unpaired *t*-test; fold change ≥ 2 or ≤ 0.5) using Euclidean distance and average linkage based on WT (BY5701; W303-1A) and F13 (F13) cells cultured in SDC medium at 25°C. The detailed data are provided in Dataset S2.

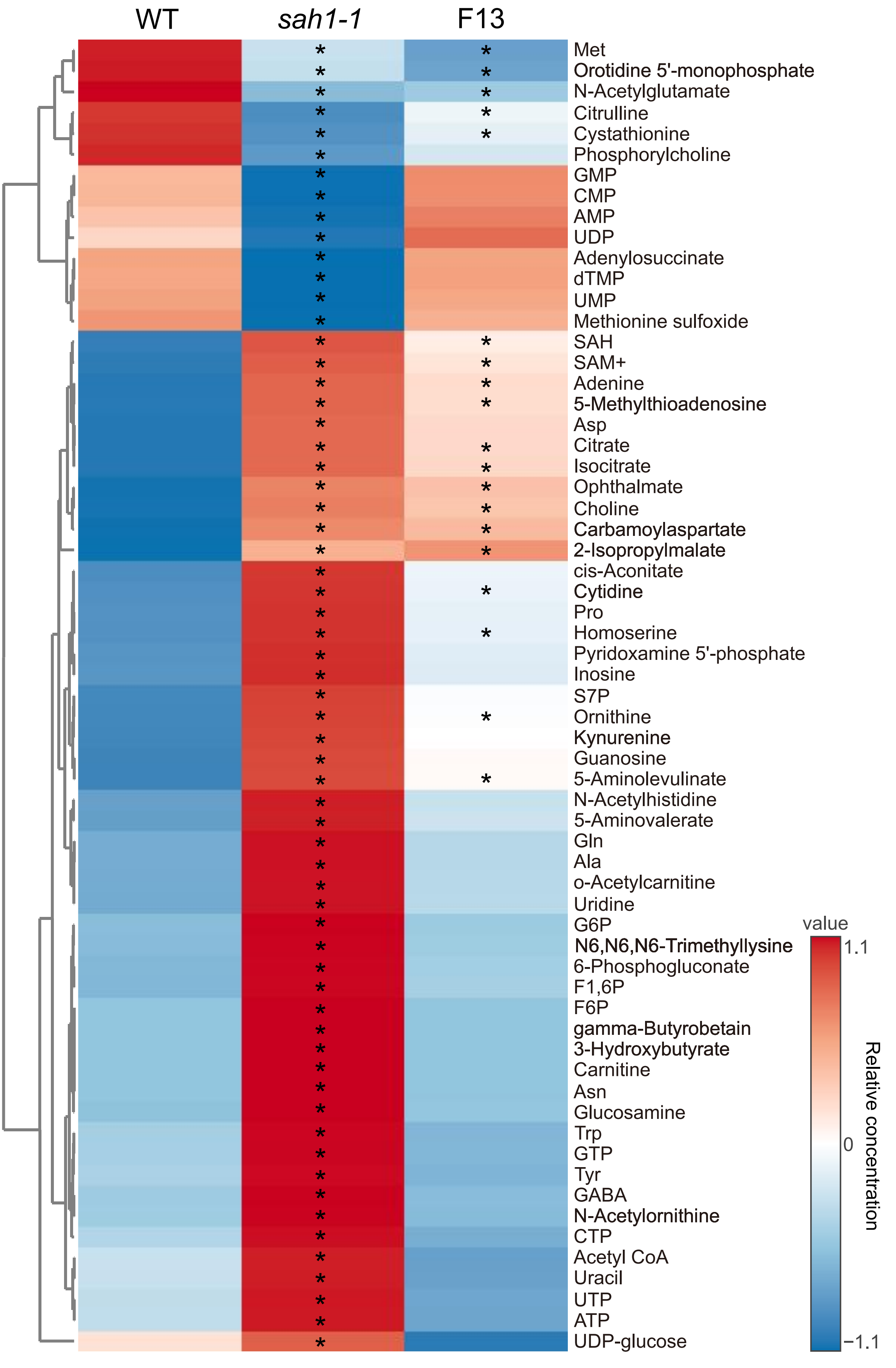

Fig. S6. Heatmap of differentially accumulated metabolites in *sah1-1* mutants and their abundance in F13 mutants, related to Figs. S4 and S5. Hierarchical clustering of altered metabolites using Euclidean distance and average linkage based on the mean of three biological replicates from WT (BY5701; W303-1A), *sah1-1* (YMM222), and F13 (F13) cells cultured in SDC medium at 25°C. Asterisks indicate metabolites with $P < 0.05$ (unpaired *t*-test) and fold change ≥ 2 or ≤ 0.5 compared to WT. The detailed data are provided in Dataset S2.

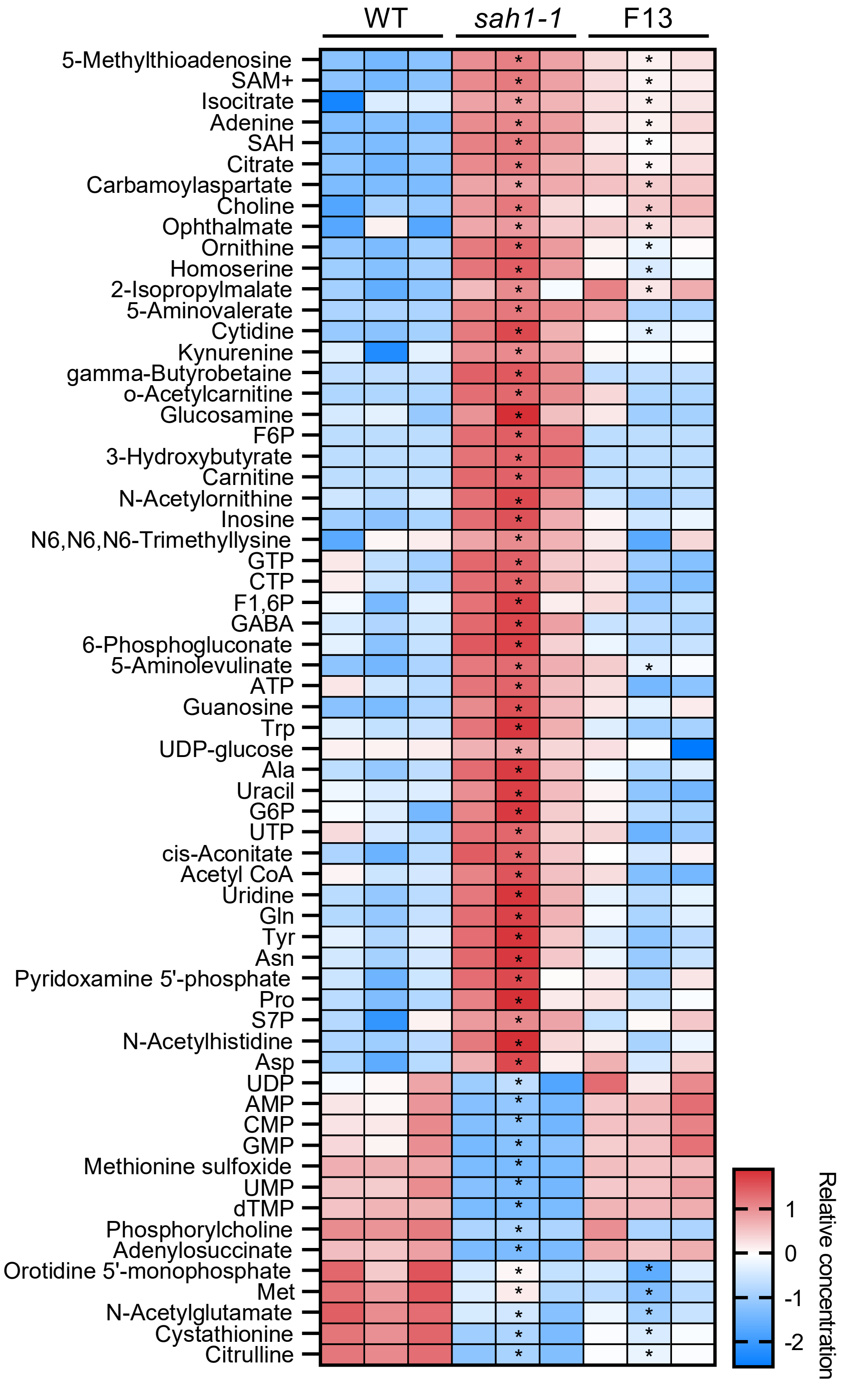

Fig. S7. Heatmap of differentially accumulated metabolites in *sah1-1* mutants and their abundance in F13 mutants, related to Figs. 2B and D. Heatmap of altered metabolites based on WT (BY5701; W303-1A), *sah1-1* (YMM222), and F13 (F13) cells cultured in SDC medium at 25°C. Asterisks indicate metabolites with $P < 0.05$ (unpaired *t*-test) and fold change ≥ 2 or ≤ 0.5 compared to WT. The detailed data are provided in Dataset S2.

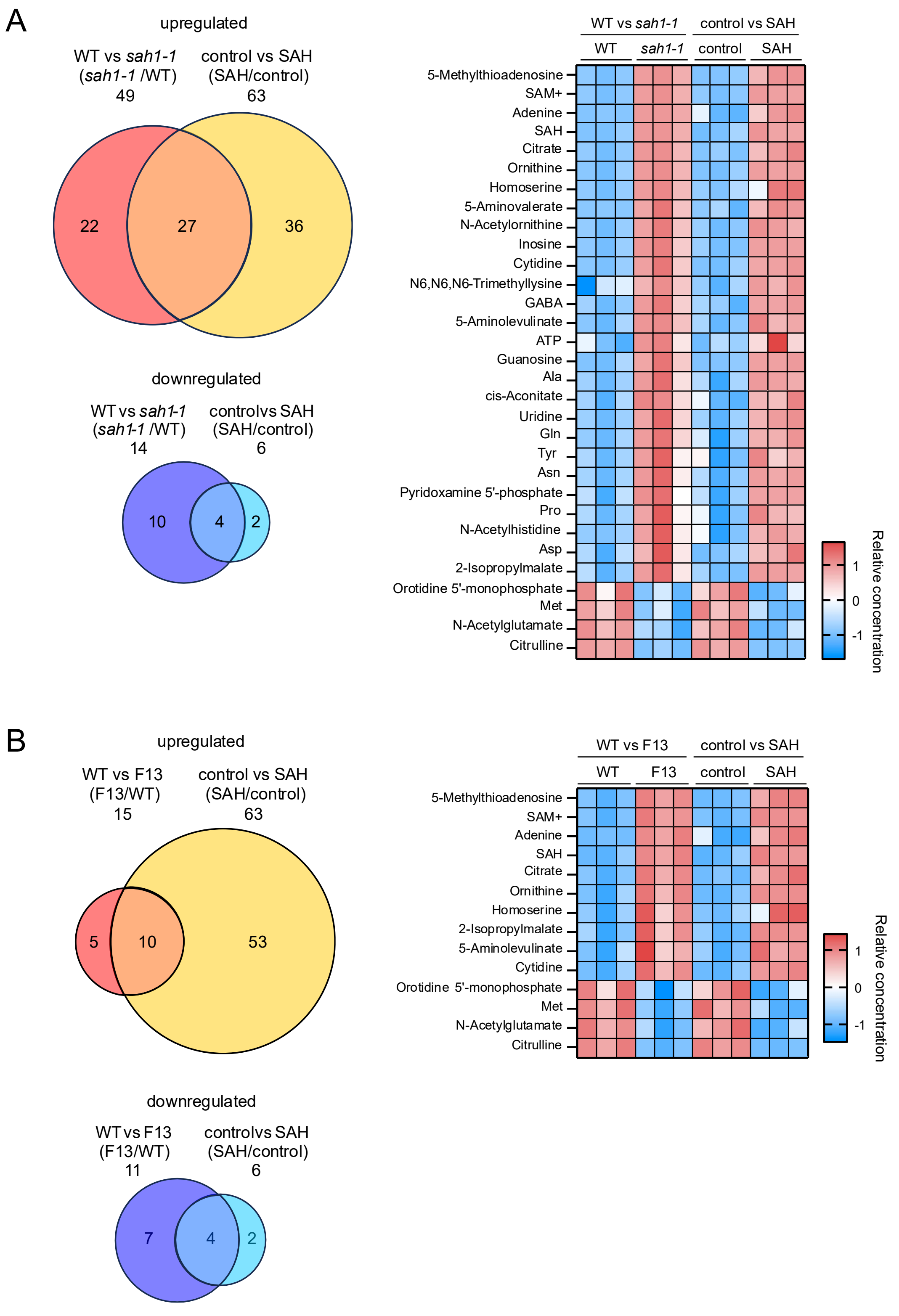

Fig. S8. Metabolomic profiles of SAH hydrolase mutants resemble those of SAH supplementation, related to Figs. 2B, D, and E. (**A**) Pie chart illustrating a comparison between the 49 metabolites that exhibited an increase in the *sah1-1* mutant strain (YMM222) and the 63 metabolites that showed an increase following SAH addition to WT (BY5701; W303-1A) cells (22) after cultivation in SDC medium at 25°C (top left). Additionally, the pie chart contrasts the 14 metabolites that decreased in the *sah1-1* mutant with the 6 metabolites that decreased after SAH addition (bottom left). The heatmap provides a summary of the metabolites that commonly increased or decreased under both conditions (right). (**B**) Pie chart presents a comparison of the 15 metabolites that increased in the F13 mutant strain (F13) with the 63 metabolites that increased following SAH addition to the WT cells (22) under the same culture conditions (top left panel). Furthermore, the pie chart compares the 11 metabolites that decreased in the F13 mutant with the 6 metabolites that decreased after SAH addition (bottom left). The heatmap summarizes the metabolites that commonly increased or decreased in both conditions (right). Statistical significance was determined using an unpaired *t*-test with FDR < 0.05. The detailed data are provided in Dataset S2.

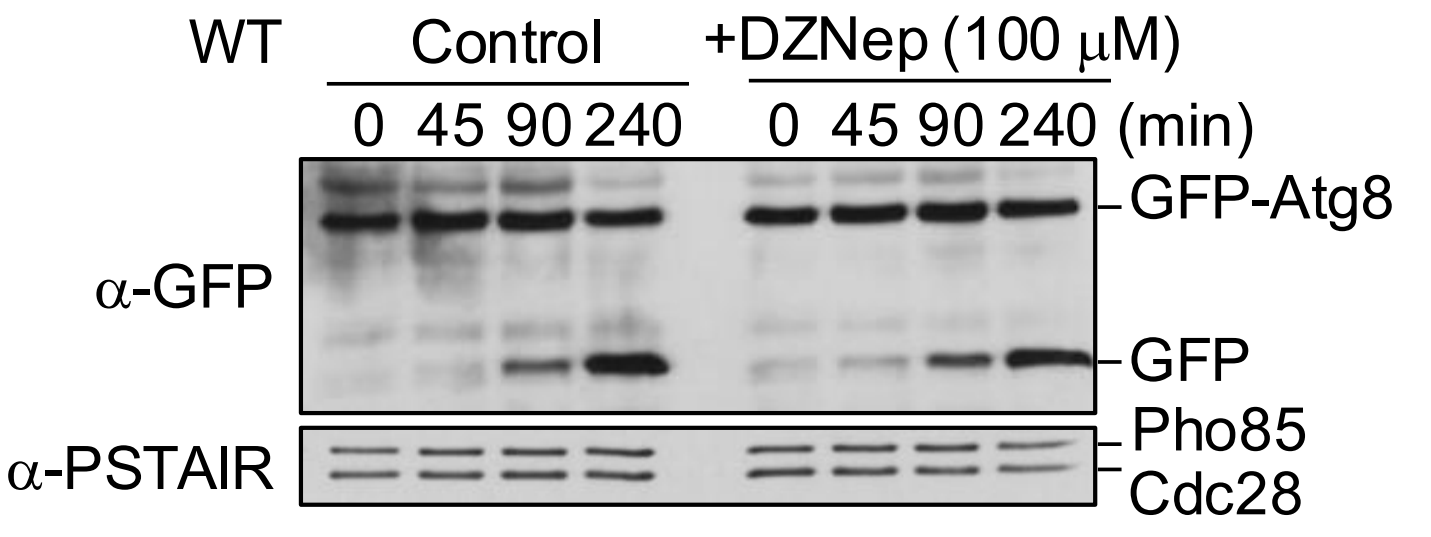

Fig. S9. Long-exposure immunoblot analysis of DZNep-induced autophagy, related to Fig. 2J. The same samples used for the 100 μM DZNep condition in the left panel of Fig. 2J were separated on a separate gel and analyzed by immunoblotting using an anti-GFP antibody. A longer exposure than that used in Fig. 2J was applied to visualize the GFP signals.

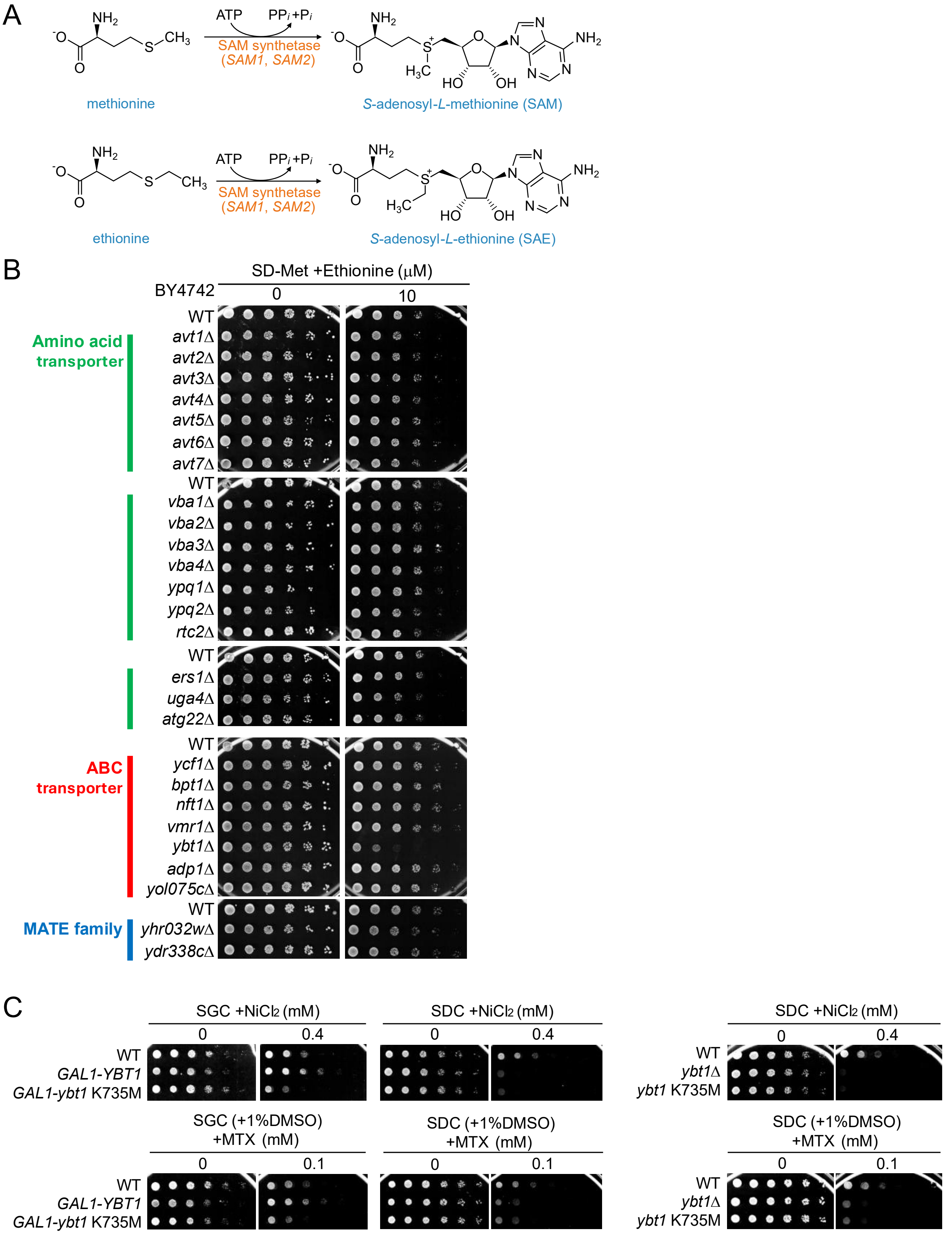

Fig. S10. Knockout of the ABC transporter *YBT1* confers ethionine sensitivity, related to Fig. 3A. (**A**) Chemical structures of SAM and SAE. (**B, C**) Serial dilution growth assays on solid SD-Met medium (B) or SDC, and SGC media (C). (B) WT (BY4742) and the indicated single-gene deletion strains, *avt1*Δ, *avt2*Δ, *avt3*Δ, *avt4*Δ, *avt5*Δ, *avt6*Δ, *avt7*Δ, *vba1*Δ, *vba2*Δ, *vba3*Δ, *vba4*Δ, *ypq1*Δ, *ypq2*Δ, *rtc2*Δ (YMA307), *ers1*Δ, *uga4*Δ, *atg22*Δ, *ycf1*Δ, *bpt1*Δ, *nft1*Δ, *vmr1*Δ, *ybt1*Δ, *adp1*Δ, *yol075c*Δ, *yhr032w*Δ, and *ydr338c*Δ, in the BY4742 background were spotted on SD-Met plates with or without 10 μM ethionine and grown at 25°C. (C) WT (BY5701; W303-1A), *GAL1-YBT1* (YMA145), and *GAL1-ybt1* K735M (YMA265) cells, as well as WT (BY5701), *ybt1*Δ (YMA241), and *ybt1* K735M (YMA280) cells, were spotted on SGC or SDC plates with or without 0.4 mM $NiCl_2$ or 0.1 mM methotrexate (MTX) in 1% DMSO at 25°C.

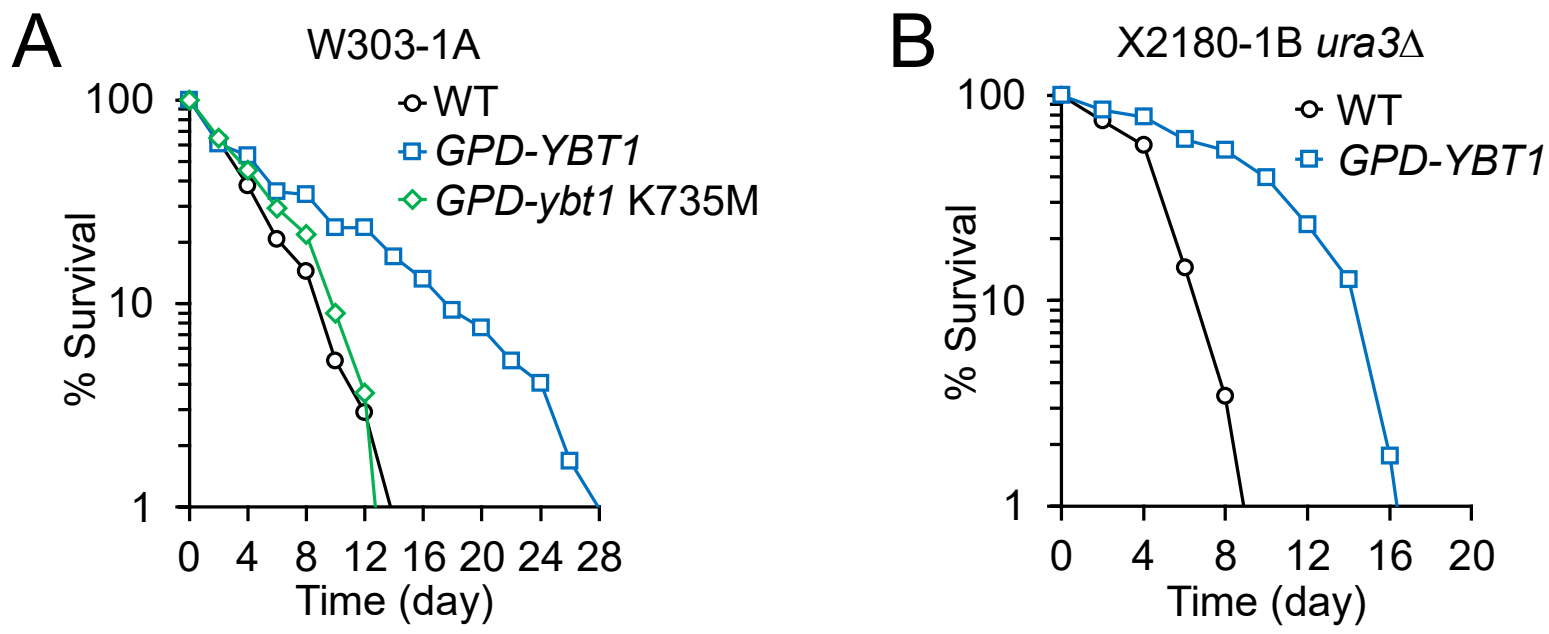

Fig. S11. *YBT1* overexpression extended CLS across diverse genetic backgrounds, related to Fig. 3C. (**A, B**) CLS survival curves (n = 3) of *YBT1*-overexpressing strains in SDC medium at 28°C, using WT (BY5701; W303-1A), *GPD-YBT1* (YMA220), and *GPD-ybt1* K735M (YMA226) cells for the W303 background (A), and WT (STY3807; X2180-1B *ura3*Δ), and *GPD-YBT1* (YMA245) cells for the X2180-1B background (B). Significance was determined using two-way ANOVA. Detailed statistics are presented in Dataset S1.
